# Epstein-Barr-Virus-Induced One-Carbon Metabolism Drives B Cell Transformation

**DOI:** 10.1016/j.cmet.2019.06.003

**Published:** 2019-09-03

**Authors:** Liang Wei Wang, Hongying Shen, Luis Nobre, Ina Ersing, Joao A. Paulo, Stephen Trudeau, Zhonghao Wang, Nicholas A. Smith, Yijie Ma, Bryn Reinstadler, Jason Nomburg, Thomas Sommermann, Ellen Cahir-McFarland, Steven P. Gygi, Vamsi K. Mootha, Michael P. Weekes, Benjamin E. Gewurz

**Affiliations:** 1 Graduate Program in Virology, Division of Medical Sciences, Harvard Medical School, 77 Avenue Louis Pasteur, Boston, MA 02115, USA; 2 Division of Infectious Diseases, Department of Medicine, Brigham and Women’s Hospital, 181 Longwood Avenue, Boston, MA 02115, USA; 3 Department of Molecular Biology and Howard Hughes Medical Institute, Massachusetts General Hospital, Boston, MA 02114, USA; 4 Department of Systems Biology, Harvard Medical School, Boston, MA 02115, USA; 5 Broad Institute of Harvard and MIT, Cambridge, MA 02142, USA; 6 Cambridge Institute for Medical Research, University of Cambridge, Hills Road, Cambridge CB2 0XY, UK; 7 Department of Cell Biology, Harvard Medical School, Boston, MA 02115, USA; 8 Department of Laboratory Medicine, West China Hospital, Sichuan University, Chengdu, Sichuan 610041, People’s Republic of China; 9 Department of Microbiology, Harvard Medical School, Boston, MA 02115, USA

**Keywords:** tumor virus, B-cell activation, virus oncoprotein, quantitative proteomics, tandem mass tag, isotope tracing, mitochondrial one-carbon metabolism, de novo serine synthesis, metabolic remodeling, folate

## Abstract

Epstein-Barr virus (EBV) causes Burkitt, Hodgkin, and post-transplant B cell lymphomas. How EBV remodels metabolic pathways to support rapid B cell outgrowth remains largely unknown. To gain insights, primary human B cells were profiled by tandem-mass-tag-based proteomics at rest and at nine time points after infection; >8,000 host and 29 viral proteins were quantified, revealing mitochondrial remodeling and induction of one-carbon (1C) metabolism. EBV-encoded EBNA2 and its target MYC were required for upregulation of the central mitochondrial 1C enzyme MTHFD2, which played key roles in EBV-driven B cell growth and survival. MTHFD2 was critical for maintaining elevated NADPH levels in infected cells, and oxidation of mitochondrial NADPH diminished B cell proliferation. Tracing studies underscored contributions of 1C to nucleotide synthesis, NADPH production, and redox defense. EBV upregulated import and synthesis of serine to augment 1C flux. Our results highlight EBV-induced 1C as a potential therapeutic target and provide a new paradigm for viral onco-metabolism.

## Highlights

• Global analysis of EBV proteome and metabolism remodeling in B cell transformation
• EBV induces mitochondrial 1C metabolic enzyme expression and flux
• EBNA2 is a viral master regulator of B cell metabolic reprogramming
• 1C drives nucleotide, mitochondrial NADPH, and glutathione production

## Graphical Abstract

**Figure:**
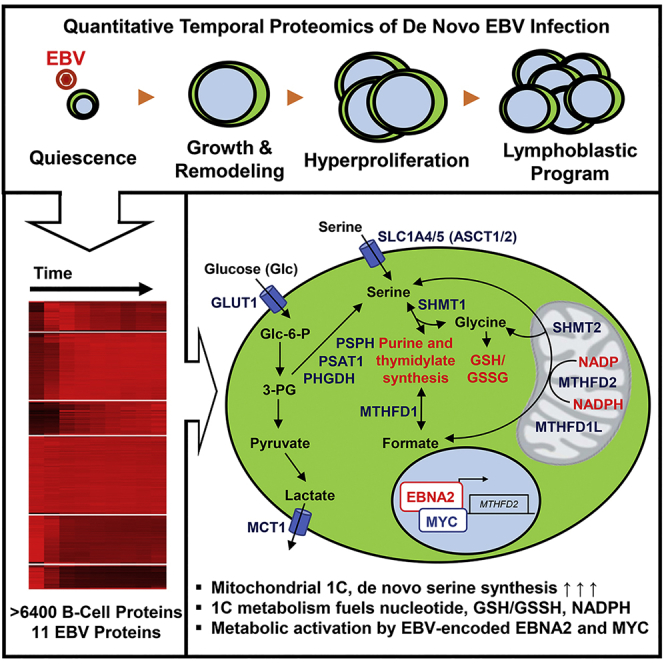

## Teaser

Global unbiased proteomic analysis reveals key metabolic pathways induced by Epstein-Barr virus critical for B cell growth transformation. In this issue of *Cell Metabolism,* Wang et al. utilized multiplexed proteomics to identify key virus-induced metabolic pathways important for outgrowth of newly infected primary human B cells. The authors describe virus-activated mitochondrial one-carbon metabolism as being crucial for nucleotide and glutathione syntheses, as well as generation of intramitochondrial NADPH.

## Context and Significance

**Epstein-Barr virus (EBV) is a herpes family virus, which is commonly associated with infectious mononucleosis (“mono” or kissing disease) and a rare number of blood cancers such as B cell lymphomas. Researchers at Cambridge University and Harvard Medical School investigated a neglected aspect of EBV infection: how EBV remodels B cell pathways to facilitate nutrient acquisition and cellular proliferation. They show that EBV infection highjacks the B cell mitochondria and the “1C folate metabolism” pathway, normally used in embryonic development, to provide the complex cellular building blocks and antioxidant support needed for cancer cell growth. The work provides an attractive rationale for developing novel folate-dependent mitochondrial 1C metabolic inhibitors for the treatment of B lymphomas.**

## Introduction

Epstein-Barr virus (EBV) is a gamma-herpes virus that successfully colonizes the B cell compartment of ∼95% of adults worldwide and was the first identified human tumor virus. EBV is the etiological agent of infectious mononucleosis (IM) and is associated with ∼1% of all human cancers worldwide, including multiple B cell malignancies (Longnecker et al., 2013) such as endemic Burkitt lymphoma (BL), Hodgkin lymphoma (HL), diffuse large B cell lymphoma (DLBCL) of the elderly, and primary central nervous system lymphoma (Shannon-Lowe et al., 2017). EBV is also the major cause of post-transplant lymphoproliferative disorder (PTLD), where viral oncoproteins drive uncontrolled B cell growth in 1%–20% of solid organ and stem-cell transplants (LaCasce, 2006, Green and Michaels, 2013).

A hallmark of EBV is its ability to transform primary human B cells into hyperproliferating blasts followed ultimately by establishment of latency, in which viral oncoproteins are expressed, but infectious virus is not produced. Through a genetically encoded viral program comprising at least three phases *in vitro* (Nikitin et al., 2010), EBV subverts major B cell activation pathways normally operative in lymph node germinal center reactions (Thorley-Lawson, 2015). First, EBV dramatically remodels B cell architecture over 72 h post-infection, where Epstein-Barr virus nuclear antigen 2 (EBNA2) and its coactivator EBNA-leader protein (EBNA-LP) act in concert to convert small quiescent cells into large activated blasts. Next, EBNA2 drives MYC expression and hyperproliferation reminiscent of BL, the fastest-growing human tumor (Molyneux et al., 2012), with mitosis every 8–12 h (Nikitin et al., 2010). Finally, EBNA2 induces expression of oncogenic EBNA3s and latent membrane proteins (LMPs). LMP1 mimics CD40 signaling to constitutively activate NF-κB (Wang et al., 2017, Kieser and Sterz, 2015), whereas LMP2A subverts the B cell receptor pathway to activate the PI3K-AKT-mTOR pathway (Cen and Longnecker, 2015). Growth transformation *in vitro* culminates in the generation of immortalized lymphoblastoid cell lines (LCLs), which serve as a major model of EBV-driven lymphoblastic lymphomas.

Each B cell transformation phase necessitates widespread remodeling of host metabolic pathways. Metabolic stress is a major barrier to EBV-induced B cell transformation; newly infected cells that fail to transform undergo growth arrest characterized by mitochondrial dysfunction and attenuated mammalian target of rapamycin (mTOR) signaling (McFadden et al., 2016). Metabolic remodeling has not been systematically investigated during EBV-driven B cell transformation or, more generally, in primary human B cell activation. While viral genes essential for B cell transformation have been identified, their global effects on B cell metabolism are poorly understood. There is little knowledge regarding the mechanisms by which EBV induces or activates key metabolic pathways to transform a quiescent B lymphocyte into a lymphoblast. Likewise, the roles of metabolic pathways in establishing and/or maintaining continual lymphoblastoid B cell growth are not well characterized.

A systematic quantitative analysis of temporal changes in host and viral proteins over the course of transformation in primary human B cells could provide a comprehensive understanding of EBV-driven metabolic reprogramming and give insights into pathways important in EBV-driven malignancies. Here, we used multiplexed tandem-mass tag (TMT)-based proteomics to measure >8,000 host proteins and 29 viral proteins over nine time points of infection of primary human B cells and in uninfected cells (Weekes et al., 2014). We found that EBV remodels B cell mitochondria and that mitochondrial one-carbon (1C) metabolism was one of the most highly induced pathways. 1C plays key roles in supporting rapid cell growth in embryonic development (Christensen and Mackenzie, 2008, Patel et al., 2005, Di Pietro et al., 2002, Patel et al., 2003), cancer (Nilsson et al., 2014), and T cell activation (Ron-Harel et al., 2016) but has not previously been studied in the context of viral oncogenesis or in primary human B cell activation.

## Results

### EBV Upregulation of Human B Cell Metabolic Pathways

**Figure 1:**
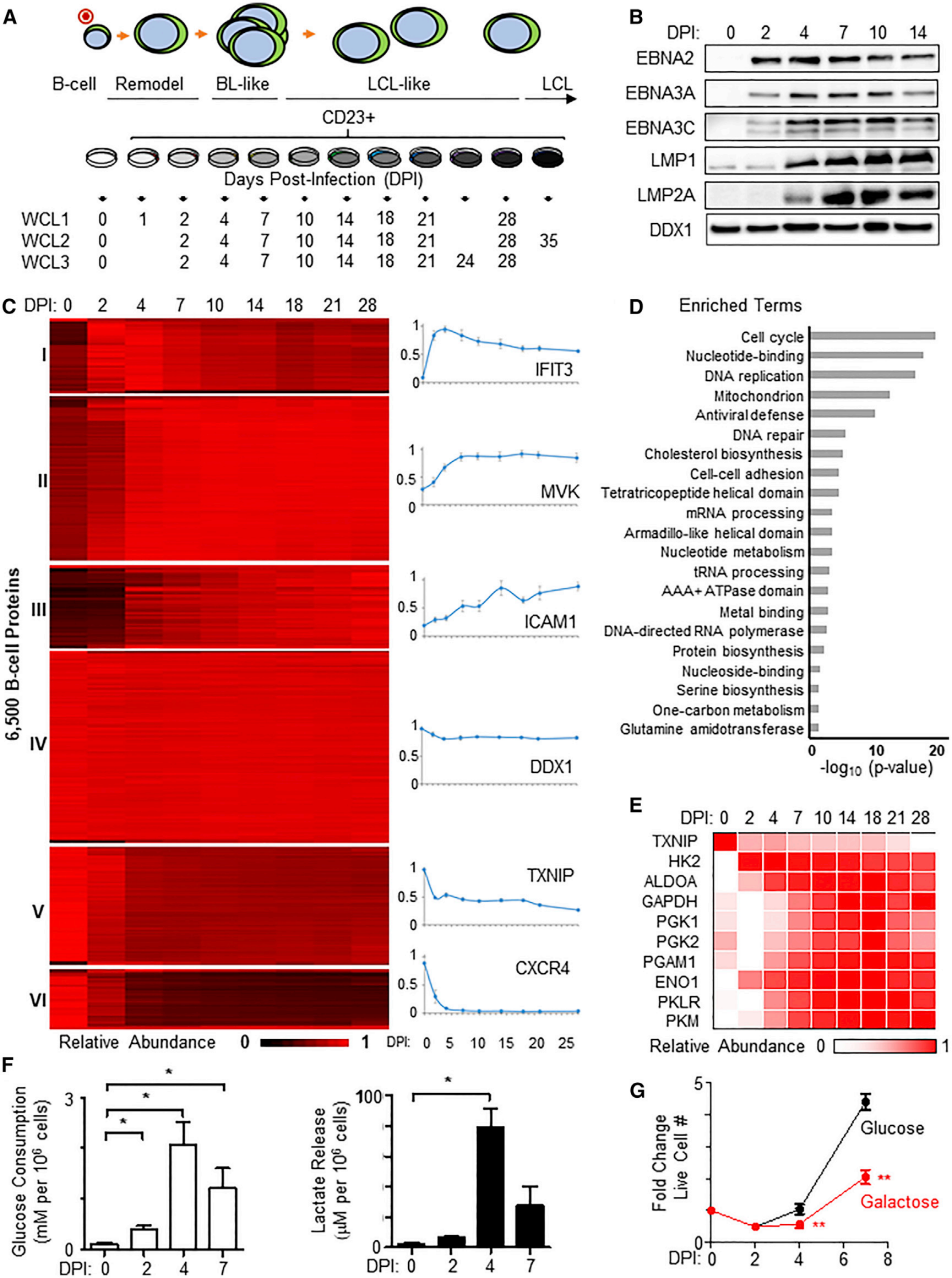
Temporal Proteomic Profiling of EBV B Cell Transformation Reveals Dramatic Metabolic Remodeling (A) Schematic of experimental workflow. Primary human B cells (3 biological replicates, each consisting of 4 independent donors) were analyzed prior to and at nine time points after EBV infection. CD23+ EBV-infected cells were enriched by fluorescence-activated cell sorting (FACS). WCL, whole-cell lysate. (B) Representative immunoblots (n = 3) of EBV nuclear antigens (EBNAs), latent membrane proteins (LMPs), and load-control DDX1 of newly infected primary B cells at the indicated days post-infection (DPI). (C) k-means clustering (k = 6) of the ∼6,500 B cell proteins quantified in all three replicates based on averaged relative abundances. Representative expression profiles are shown to the right of each k-means cluster. To determine the actual number of distinct classes of host protein expression, the k-means approach was used with 1–12 classes to cluster viral proteins, and the summed distance of each protein from its cluster centroid was calculated (see Figure S1D). (D) Functional enrichment within all proteins upregulated >2-fold with p < 0.075 4 days post-EBV infection (clusters I–III) against a background of all quantified proteins. Overall, 41 clusters were significantly enriched (Table S2). For the purposes of simplified display, these were concatenated into hierarchical parent terms, where available for individual clusters that had been identified using Uniprot or Gene Ontology. For example, “Sister chromatid cohesion,” “DNA condensation,” and “Kinesin, motor domain” were concatenated into “Cell cycle.” (E) Heatmap of averaged relative abundances of glycolytic pathway component enzymes at each indicated time point. (F) LC-MS analysis of media glucose consumption and lactate production from cultures of primary B cells at the indicated time points post-infection. The 24-h mean ± SEM decrease in media glucose and 24-h increase in media lactate are shown at the indicated DPI, n = 3. ^∗^p < 0.05 (paired one-tailed t test). (G) Growth curves of newly infected primary human B cells grown in complete media containing either glucose or galactose. Data show the mean ± SEM, n = 3. ^∗∗^p < 0.01 (paired one-tailed t test). See also Figures S1–S3 and Tables S1 and S2.

To identify virus-induced metabolic pathways important for EBV-driven B cell growth, we used 10-plex TMT and MS3 mass spectrometry to analyze primary human CD19+ B cells either left uninfected or infected at a low multiplicity with the B95-8 strain of EBV, which was originally isolated from a patient with IM. Successfully infected B cells were isolated by flow cytometry at nine time points after initial infection using CD23 plasma membrane (PM) expression as a proxy for infection (Wang et al., 1987, Thorley-Lawson and Mann, 1985). Three whole-cell lysate (WCL) biological replicates, each comprising cells pooled from four distinct human donors, were performed (Figure 1A). We additionally quantified changes in PM protein expression for one replicate. Immunoblots demonstrated the expected pattern of EBV oncoprotein expression (Figure 1B).

We quantified 8,054 B cell and 29 EBV-encoded proteins in at least one replicate. Across all three replicates, 6,455 B cell and 11 EBV-encoded proteins (Figures 1C and S1A) were quantified; data exhibited strong concordance across replicates (Figures S1B and S1C). All data are shown in Table S1, where the worksheet “Plots” is interactive and allows generation of temporal graphs of WCL and PM expression of any of the identified human and viral proteins. Throughout this manuscript, all analyses are based on the average values from all analyzed replicates.

Six clusters of B cell proteins with distinct temporal expression patterns over the transformation time course were identified by way of k-means analysis (Figures 1C and S1D). We used Database for Annotation, Visualization, and Integrated Discovery (DAVID) software (Huang da et al., 2009a, Huang da et al., 2009b) to identify pathways enriched among significantly upregulated proteins (clusters I–III) at 4 days post-infection (DPI) and identified multiple metabolic terms including 1C metabolism and serine biosynthesis (Figure 1D). Among quantified viral proteins, particularly high EBNA2 expression was observed 2 DPI, which correlated with markedly elevated levels of MYC, a well-known master regulator of cellular metabolism (Figures S2A and S2B). We also discovered two new EBV open reading frames, whose expressed gene products increased late in infection (Figures S2A, S2C, and S2D). Approximately 20 EBV lytic cycle proteins were also quantified, including several metabolic enzymes (Figure S2A). This likely represents leaky lytic protein expression rather than true lytic replication, as we were able to detect 72 EBV proteins in our similar TMT analysis of the EBV B cell lytic cycle (Ersing et al., 2017). Alternatively, it might have been due to full or abortive lytic replication in a small population of cells. However, flow cytometric analysis at 4 DPI identified that most cells were negative for gp350, indicating that lytic reactivation was unlikely to be the driver of the metabolic changes (Figures S2E and S2F). Profiling of 712 PM proteins revealed widespread EBV-driven remodeling of the PM proteome, particularly amino acid and ion transporters, coinciding with the onset of B cell hyperproliferation (Figures S3A–S3C).

### Early Induction of Aerobic Glycolysis in Newly Infected B cells

EBV induces aerobic glycolysis in infected B cells, but how early this occurs in B cell transformation remains undefined (McFadden et al., 2016, Darekar et al., 2012, Sommermann et al., 2011). Early upregulation of all glycolytic enzymes was detected, with the rate-limiting enzyme hexokinase 2 (HK2) highly induced by 2 DPI (Figure 1E). Thioredoxin-interacting protein (TXNIP), a potent negative regulator of glucose metabolism (Parikh et al., 2007), was concomitantly and strongly downregulated by EBV (Figure 1E). PM proteomic data and subsequent validation by flow cytometry suggested that EBV infection induced substantial re-localization of GLUT1 to the PM (Figure S3D). Consistent with GLUT1 trafficking to the PM and subsequent enhanced glycolytic flux, B cell glucose consumption and lactate release were increased by 2 DPI and were maximal at 4 DPI (Figures 1F and S3E). EBV-driven outgrowth was strongly impaired when cells were cultured in media containing galactose instead of glucose (Figure 1G), highlighting glucose as a key carbon source in viral B cell transformation. As EBNA2 is the major EBV transcription factor expressed at this early time point, we determined whether it was necessary for EBV-driven aerobic glycolysis. We made use of the 2-2-3 EBNA2-HT B cell line (hereafter referred to as EBNA2-HT), where 4-hydroxytamoxifen (4HT) positively regulates nuclear localization and stability of a conditional EBNA2 allele, comprising EBNA2 fused to a mutant estrogen-receptor-ligand-binding domain (Zhao et al., 2006, Schuhmacher et al., 2001). Conditional inactivation of EBNA2-HT by 4HT withdrawal strongly impaired LCL lactate release (Figure S3F), further suggesting an important role for EBNA2 in stimulating glycolysis.

### EBV Triggers Extensive Mitochondrial Remodeling in Newly Infected B Cells

**Figure 2:**
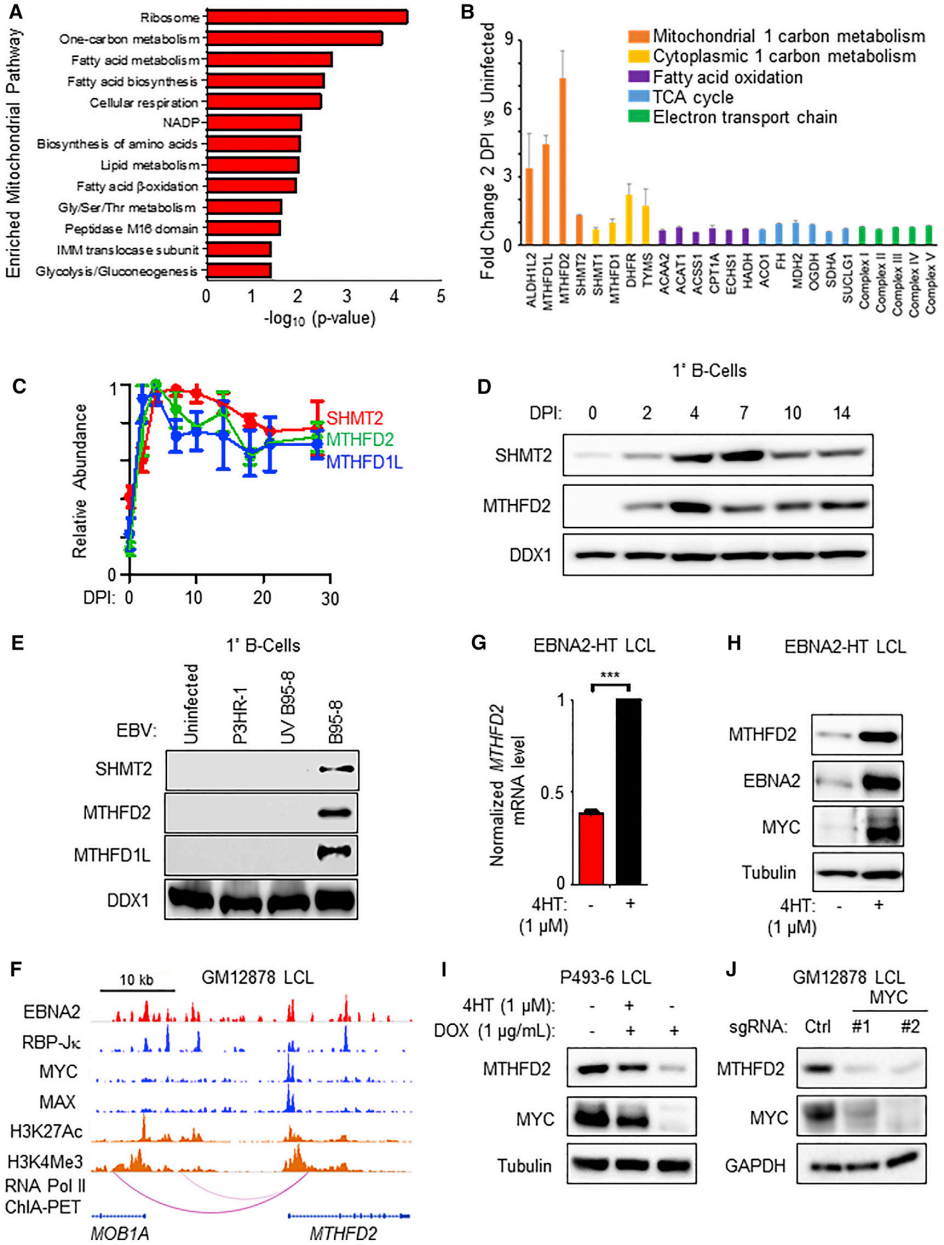
EBV Induces Mitochondrial One-Carbon Metabolism in Newly Infected B Cells (A) Functional enrichment of mitochondrial pathways most highly induced by primary human B cell upon EBV infection. Enrichment was examined in the subset of proteins upregulated by at least >2-fold, at ≥1 time point, in comparison to all quantified mitochondrial proteins. Representative terms are shown, and full details of enriched terms are shown in Table S3. (B) Fold change in expression of select metabolic enzymes at 2 DPI relative to resting B cells. Data show the mean + SD, n = 3. (C) Temporal plots of relative abundances of the indicated mitochondrial one-carbon enzymes at the indicated DPI. Data show the mean ± SEM, n = 3. (D) Immunoblot analysis of SHMT2, MTHFD2, or load-control DDX1 expression in primary human B cells at the indicated DPI. Representative of n = 3. (E) Immunoblot analysis of SHMT2, MTHFD2, MTHFD1L, or DDX1 expression in uninfected cells or cells equally infected with either P3HR-1, UV-inactivated B95-8, or B95-8 virus 4 DPI. n = 2. (F) ChIP-seq tracks of the indicated transcription factors or the activating histone epigenetic marks H3K27Ac or H3K4Me3 at the GM12878 LCL *MTHFD2* locus. Shown also are GM12878 ChIA-PET-defined long-range DNA linkages between an upstream *MOB1A* locus enhancer and *MTHFD2*. (G) Quantitative PCR analysis of *MTHFD2* transcript expression in EBNA2-HT cells cultured in the presence (permissive for growth) or absence (non-permissive for growth) of 4HT (1 μM) for 48 h. Data show the mean + SEM, n = 3. ^∗∗∗^p < 0.005 (one-sample t test). (H) Immunoblot analysis of MTHFD2, MYC, and tubulin expression in EBNA2-HT cells cultured in the presence or absence of 4HT (1 μM). Representative of n = 3. (I) Immunoblot analysis of MTHFD2, MYC, and tubulin expression in P493-6 cells cultured with the indicated supplement. Representative of n = 3. (J) Immunoblot analysis of MYC, MTHFD2, and GAPDH expression in Cas9+ GM12878 LCLs following the expression of non-targeting control or independent *MYC*-targeting sgRNAs. n = 2. See also Figure S4 and S5 and Table S3.

Relatively little is known about EBV-mediated reprogramming of the mitochondrion. Using the MitoCarta 2.0 database (Calvo et al., 2016), 799 B cell mitochondrial proteins were identified by our temporal profiling. Mitochondrial proteome remodeling commenced shortly after infection and even prior to mitosis, which is suggestive of an important role in viral B cell growth transformation (Figures 1D, 2A, and S4A–S4C). Notably, since most mitochondrial proteins are encoded by the host cell nuclear genome, EBV nuclear antigens can regulate their expression.

EBV upregulated 35 of the 98 quantified nuclear genome-encoded electron transport chain (ETC) components (Figure S4B) by ≥1.5-fold at ≥one time point, including two ETC components encoded by the mitochondrial genome, MT-ATP6 and MT-ATP8. We observed enhanced basal and maximal oxygen consumption rates (OCRs) in newly infected cells (Figure S4D), suggesting that increased oxidative capacity might be important for successful outgrowth. Newly infected cells were treated with either piericidin A, a complex I inhibitor, or antimycin, a complex III inhibitor. Each significantly diminished EBV-driven B cell outgrowth (Figure S4E). Thus, the concurrent induction of the Warburg effect and the upregulation of oxidative phosphorylation are each important for supporting outgrowth of B cells undergoing viral transformation.

DAVID analysis identified ribosome biogenesis and 1C metabolism as the most strongly upregulated mitochondrial pathways in newly infected B cells (Figures 2A and 2B). Mitochondrial 1C metabolism uses serine as a precursor for 1C unit, NAD(P)H, ATP and glycine generation. EBV robustly upregulated mitochondrial 1C enzymes by 2 DPI (Figures 2A–2D), whereas relative abundances of cytoplasmic 1C, mitochondrial fatty acid oxidation, and tricarboxylic acid cycle (TCA) proteins showed little change (Figure 2B). EBV also upregulated the mitochondrial folate transporter SLC25A32 (Figure S4A), dihydrofolate reductase (DHFR) (Figure 2B), which is responsible for generating the 1C carrier tetrahydrofolate (THF) and is a target of the antifolate drug methotrexate, and the recently identified mitochondrial serine transporter SFXN1 (Kory et al., 2018) (Table S1). Notably, EBV upregulated MTHFD2, a mitochondrial enzyme not expressed by resting B cells or by most adult cells but among the most highly induced metabolic enzyme in human cancer (Nilsson et al., 2014). Collectively, these data suggest that EBV remodels B cell mitochondria to support the substantial physiological shift from quiescence to rapid lymphoblastic proliferation.

### EBNA2 Upregulates Mitochondrial 1C Metabolism

ATF4 is the major mitochondrial 1C gene transcription activator (Ben-Sahra et al., 2016, Bao et al., 2016). Although *ATF4* is transcribed in LCLs (Arvey et al., 2012), ATF4 protein was not detected by proteomics or by immunoblot. In support of this result, we validated the ATF4 antibody by showing that LCL ATF4 immunoblot signal could be induced by tunicamycin treatment and that this signal could be suppressed by CRISPR ATF4 targeting. Notably, neither tunicamycin-mediated ATF4 induction nor CRISPR-mediated ATF4 depletion altered LCL MTHFD2 levels (Figure S5A). Likewise, leaky EBV lytic gene expression was unlikely to drive 1C induction since our prior proteomic analysis of B cell EBV lytic reactivation (Ersing et al., 2017) did not demonstrate 1C enzyme induction (Figure S5B).

Given the key EBNA2 role in aerobic glycolysis induction, we hypothesized that EBNA2 might be a viral master regulator of B cell metabolism and tested its role in mitochondrial 1C induction. Primary human B cells were equally infected with B95-8 or the non-transforming P3HR-1 EBV strains (Miller et al., 1974, Miller et al., 1975) (Figures S5C and S5D), using input viral genome copy number to normalize level of infection. P3HR-1 lacks EBNA2 and most of the EBNA-LP open reading frames (Rowe et al., 1985, Rymo et al., 1985, Tsang et al., 1991, Wang et al., 1990). B95-8, but not P3HR-1 or UV-irradiated B95-8, could induce the expression of mitochondrial 1C enzymes at 4 DPI (Figure 2E) despite equivalent infection levels, as evaluated by post-infection viral genome copy number assay and EBNA1 confocal immunofluorescence analysis. These data suggest that expression of EBV-encoded EBNA2 and/or EBNA-LP, rather than an innate immune response to the viral particle, is required for the induction of mitochondrial 1C metabolism.

To further investigate possible EBNA2 roles in MTHFD2 induction, we used publicly available LCL chromatin immunoprecipitation with deep sequencing (ChIP-seq) (ENCODE Project Consortium, 2012, Wood et al., 2016, Zhao et al., 2011) and chromatin interaction analysis by paired-end tag (ChIA-PET) data (Jiang et al., 2017) to identify transcription factor occupancy. EBNA2 and its cofactor RBP-Jκ were found to co-occupy the *MTHFD2* promoter, as well as upstream intragenic and intergenic enhancers that loop to the *MTHFD2* promoter (Figure 2F). Inactivation of the conditional EBNA2 allele resulted in rapid loss of MTHFD2 transcript and protein (Figures 2G and 2H).

EBNA2 highly upregulates MYC (Kaiser et al., 1999, Zhou et al., 2015), and both proteins can act synergistically to induce EBV targets. As expected, conditional EBNA2 inactivation caused rapid downregulation of MYC levels (Figure 2H). Since MYC and MAX co-occupy the LCL *MTHFD2* promoter (Figure 2F) and given MYC’s role in MTHFD2 regulation in acute myelogenous leukemia (Pikman et al., 2016), we investigated a potential role for MYC in EBV induction of MTHFD2 expression. With the P493-6 B cell line, an LCL that expresses a conditional 4HT-responsive EBNA2 allele and also carries a heterologous MYC allele controlled by a Tet-OFF promoter (Schuhmacher et al., 2001), we found that re-expression of MYC was sufficient to restore MTHFD2 expression upon EBNA2 inactivation by 4HT withdrawal (Figure 2I) and observed that MTHFD2 and MYC levels were closely correlated. Cas9 editing of *MYC* directed by either of two distinct single-guide RNAs (sgRNAs) caused loss of MYC protein and a concomitant decrease in MTHFD2 protein abundance prior to LCL cell death (Figure 2J). Taken together, these results identify EBNA2 as the first viral oncoprotein important for mitochondrial 1C metabolism induction by a mechanism that involves MYC instead of ATF4.

### EBNA2 and MYC Upregulate B Cell Import and Mitochondrial 1C Catabolism of Serine

**Figure 3:**
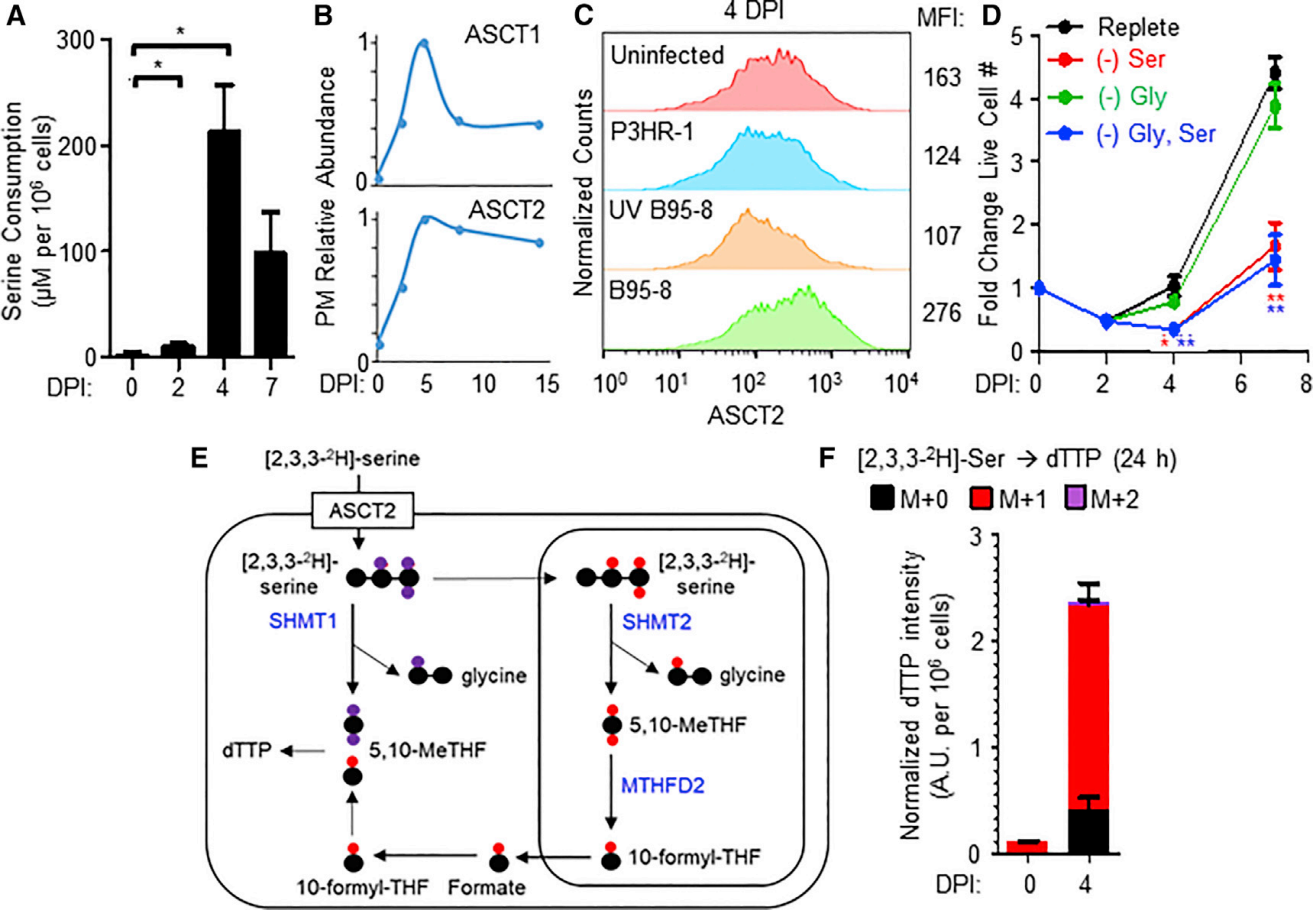
EBV Infection Induces B Cell Serine Transporter Expression, Uptake, and Catabolism (A) LC-MS measurements of media serine concentrations of primary B cells cultured at the indicated DPI. Values indicate mean + SEM serine consumption over a 24-h period at the indicated time point, n = 3. ^∗^p < 0.05 (paired two-tailed t test). (B) Temporal plots of PM relative abundances of the neutral amino acid transporters ASCT1 and ASCT2. (C) Flow cytometry of ASCT2 in uninfected B cells maintained in culture for 4 days or at 4 DPI with the indicated EBV strain at equal levels of infection. Representative of n = 3. (D) Growth curves of newly infected primary B cells cultured in either replete media or media lacking serine, glycine or both serine and glycine. Data show the mean ± SEM, n = 3. ^∗^p < 0.05; ^∗∗^p < 0.01 (paired one-tailed t test). (E) Schematic illustrating the fate of [2,3,3-^2^H]-serine in either the cytosolic (purple) or mitochondrial (red) one-carbon metabolic pathway. Enzymes involved in catabolizing serine are indicated in blue bold font. (F) LC-MS measurements of dTTP isotopologues from 0 and 4 DPI cells grown in the presence of [2,3,3-^2^H]-serine. Data show the mean with SEM, n = 3. See also Figures S5 and S6.

Serine and glycine fuel mitochondrial 1C metabolism (Ducker and Rabinowitz, 2017, Yang and Vousden, 2016). We hypothesized that newly infected cells might therefore increase uptake and/or *de novo* synthesis of serine or glycine. Media liquid chromatography-mass spectromtry (LC-MS) analysis revealed avid consumption of serine but not glycine over the first week after EBV infection, which peaked at 4 DPI, coinciding with the onset of BL-like hyperproliferation and maximal MYC abundance (Figures 3A and S5E). Consistent with this observation, EBV markedly upregulated whole cell and PM levels of the major serine transporters SLC1A4 (ASCT1) and SLC1A5 (ASCT2) by 4 DPI (Figures 3B and S5F). In contrast, neither EBNA2-deficient P3HR-1 nor UV-irradiated B95-8 EBV robustly upregulated ASCT2 (Figure 3C).

Serine and glycine are non-essential amino acids but are often important for transformed cell growth (Locasale et al., 2011, Possemato et al., 2011). To test whether EBV infection renders primary B cells auxotrophic for serine and/or glycine, we performed growth transformation assays in either replete media or media deficient for serine and/or glycine. Interestingly, while glycine withdrawal had little effect, exogenous serine depletion significantly impaired EBV-driven primary B cell outgrowth (Figure 3D). The lack of effect with glycine withdrawal was consistent with the notion that serine catabolism generates sufficient intracellular glycine (Ducker et al., 2017). These results support a model where EBNA2 upregulates import of exogenous serine to enable B cell metabolic remodeling and hyperproliferation.

Imported serine can be catabolized by either the cytosolic or mitochondrial 1C pathways. To identify the 1C compartment activated by EBV infection, we performed isotope tracing experiments on newly infected primary human B cells grown in the presence of [2,3,3-^2^H]-serine. The cytosolic pathway results in the stable incorporation of two deuterons into deoxythymidylate triphosphate (M+2 labeling of dTTP), whereas mitochondrial 1C results in the production of M+1-labeled dTTP (Figure 3E). Isotope tracing identified M+1 dTTP as the predominant species (Figure 3F), indicating that EBV activates mitochondrial 1C metabolism in newly infected cells and that a major reason that EBV upregulates the import of exogenous serine is to support mitochondrial 1C metabolism.

### EBNA2 and MYC Upregulate *De Novo* Synthesis of the 1C Fuel Serine

In addition to EBV’s effects on serine import, temporal proteomic analysis also highlighted EBV upregulation of the *de novo* serine synthesis (DNSS) pathway, which converts the glycolytic intermediate 3-phosphoglycerate (3-PG) into serine (Figure S5G). All DNSS enzymes were strongly induced by EBV by 4 DPI (Figures S6A and S6B). Given the finding that EBV infection induces serine auxotrophy, we next tested whether DNSS was also important for infected cell growth. Newly infected cells and GM12878 LCLs were treated with either DMSO or with one of two structurally distinct PHGDH inhibitors, CBR-5884 (Mullarky et al., 2016a, Mullarky et al., 2016b) or NCT-503 (Pacold et al., 2016b, Pacold et al., 2016a). PHGDH inhibition by either antagonist significantly diminished B cell proliferation (Figures S6C and S6D).

To investigate EBV’s effects on serine flux, isotope tracing with U^13^C-glucose was performed with resting and newly infected B cells (Figure S6E). M+3 labeled serine was detected in newly infected cells, while resting cells showed no detectable signal, suggesting increased DNSS from U^13^C-glucose-derived 3-PG and consistent with the notion that EBV activates DNSS flux (Figure S6F). Treatment of newly infected cells with either CBR-5884 or NCT-503 resulted in significant decreases in cellular and media M+3 serine levels, indicative of the inhibitors’ on-target effects (Figure S6F). Cellular M+3 serine abundance was relatively low (∼1%) in newly infected cells grown under replete conditions, perhaps because DNSS-derived serine may be rapidly consumed. However, when serine was excluded from the media and formate was supplemented, the proportion of M+3 serine increased to nearly 15% (Figure 6G). This finding is consistent with the hypothesis that DNSS may serve to augment intracellular serine pools under conditions of limiting extracellular serine, as might happen *in vivo.*

Further supporting the role of EBNA2 as a viral master regulator of B cell metabolism, its conditional inactivation of EBNA2 in the EBNA2-HT cell line impaired *PHGDH* mRNA expression (Figure S6H). In addition, conditional MYC expression in P493-6 B cells grown under EBNA2-non-permissive conditions was sufficient to upregulate mRNAs encoding 1C and DNSS enzymes (Figure S6I) (Lin et al., 2012). Collectively, our data suggest that EBNA2- and MYC-induced serine uptake and synthesis are important determinants of EBV-infected B cell proliferation.

### Serine Catabolism and Mitochondrial 1C are Critical for EBV-Infected B Cell Growth

**Figure 4:**
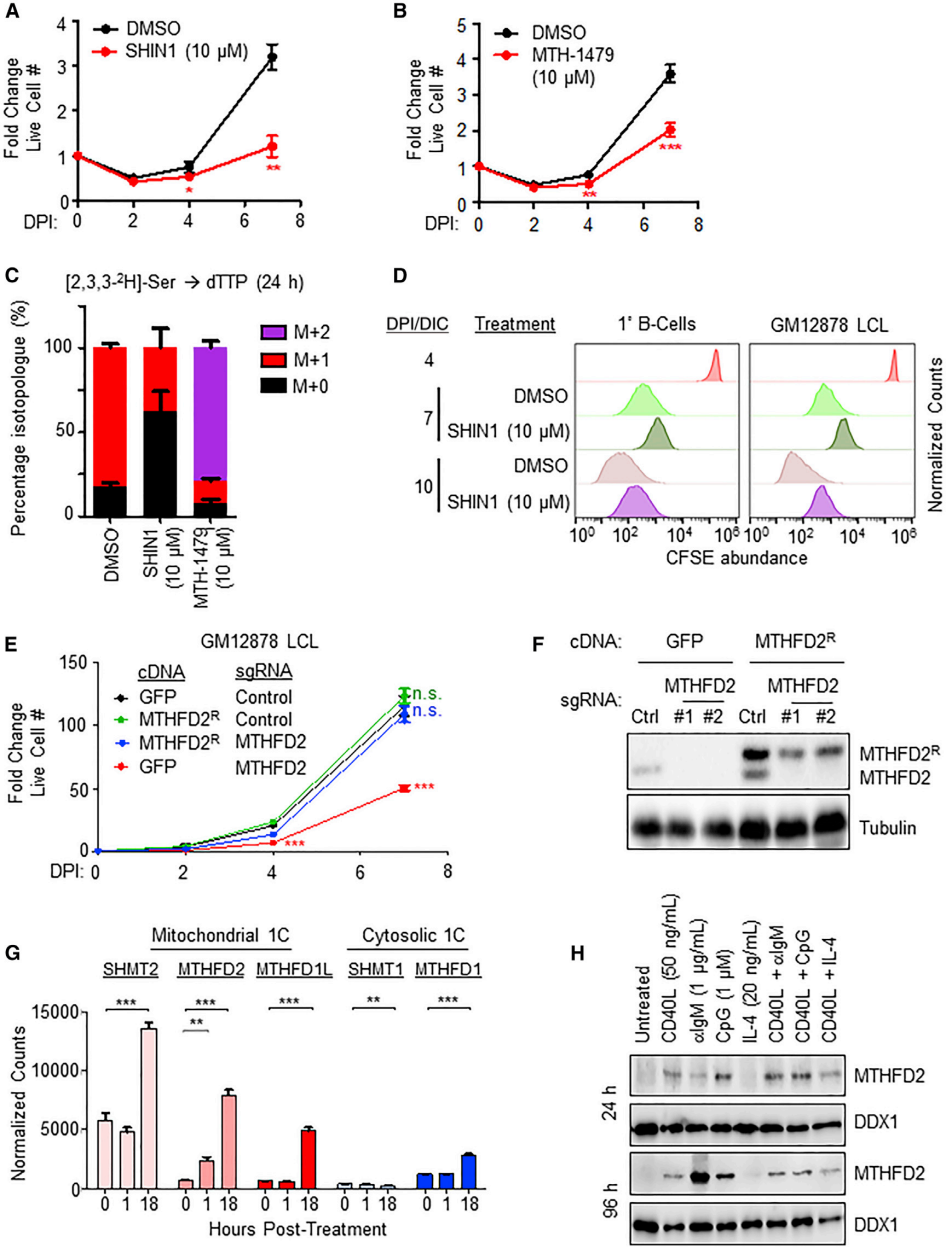
One-Carbon Metabolism Is Crucial for Efficient EBV-Infected Cell Growth and Survival (A) Growth curves of newly infected primary B cells treated with DMSO or SHIN1 (10 μM). Data show the mean ± SEM, n = 4. ^∗∗^p < 0.01 (paired one-tailed t test). (B) Growth curves of newly infected B primary cells treated with DMSO or MTH-1479 (10 μM). Data show the mean ± SEM, n = 7. ^∗∗∗^p < 0.005 (paired one-tailed t test). (C) LC-MS measurements of dTTP isotopologues from 4 DPI cells treated for 24 h with either DMSO, SHIN1 (10 μM) or MTH-1479 (10 μM), and grown in the presence of [2,3,3-^2^H]-serine. Data show the mean with SEM, n = 3. (D) CFSE dye dilution assays of newly infected primary B cells and LCLs treated with either DMSO or SHIN1 (10 μM). Cells were stained with CFSE at 4 DPI and immediately treated with either DMSO or SHIN1. Data shown are representative of n = 3. (E) Growth curve analysis of Cas9+ GM12878 LCL following expression of the indicated *GFP* control or *MTHFD2*^R^ rescue cDNAs and the indicated control or *MTHFD2*-targeting sgRNAs. ^∗∗∗^p < 0.005 (paired one-tailed t test). (F) Immunoblot analysis of WCL from Cas9+ GM12878 LCL following expression of the indicated *GFP* control or *MTHFD2*^R^ rescue cDNAs and the indicated control or *MTHFD2*-targeting sgRNAs. Representative blot of n = 3 replicates shown. (G) Normalized RNA-seq counts of the indicated mRNAs encoding 1C enzymes in primary human B cells stimulated by MEGACD40L (50 ng/mL) for the indicated times. ^∗^p < 0.05; ^∗∗^p < 0.01; ^∗∗∗^p < 0.005 (unpaired two-tailed t test). (H) Immunoblot analysis of WCL from primary human B cells stimulated as indicated. Representative of n = 2. See also Figure S7.

To test whether EBV-induced 1C metabolism was important for transforming B cell outgrowth, newly infected primary B cells were treated with one of two chemically distinct 1C pathway inhibitors. SHIN1 selectively blocks cytosolic SHMT1 and mitochondrial SHMT2 (Ducker et al., 2017), while MTH-1479 is a specific MTHFD2 antagonist (Chandrasekaran et al., 2017). Inhibition of either SHMT1/2 or MTHFD2 significantly diminished EBV-driven primary B cell proliferation (Figures 4A and 4B). We confirmed the on-target effects of SHIN1 and MTH-1479 by LC-MS analysis of dTTP from cells fed [2,3,3-^2^H]-serine and treated with either inhibitor. As expected for on-target effects, SHIN1 treatment resulted in the loss of M+1-labeled dTTP and an increase in the unlabeled M+0 population, while MTH-1479 treatment caused a decrease in M+1 dTTP with a concomitant, large increase in the M+2 isotopologue, consistent with reversal of 1C flux (Figure 4C).

To further assess 1C roles in EBV-driven proliferation and survival, we treated newly infected primary B cells and GM12878 LCL with SHIN1 and performed carboxyfluorescein diacetate succinimidyl ester (CFSE) dye dilution and 7-aminoactinomycin D (7-AAD) viability assays. SHIN1 decreased proliferation and increased cell death of both newly infected B cells and fully transformed GM12878 LCLs (Figures 4D and S7A). To exclude the possibility that these proliferative defects were due to off-target effects of SHIN1, we attempted to rescue SHIN1-treated cells with formate supplementation. Addition of millimolar levels of formate to the culture media rescued the growth of newly infected cells; addition of excess glycine did not promote further growth (Figure S7B). Furthermore, SHIN1 negatively impacted outgrowth of primary human B cells in an *in vitro* transformation assay (Figure S7C). To further investigate EBV-induced mitochondrial 1C roles in lymphoblastoid B cell growth and survival, Cas9-expressing GM12878 LCLs were transduced with lentivirus expressing non-targeting control or an *MTHFD2* targeting sgRNA. MTHFD2 knockout (KO) by either of two independent sgRNAs significantly diminished LCL proliferation (Figure S7D) and caused accumulation of cells at the G1/S phase (Figure S7E). Stable expression of a Cas9-resistant silent point mutant MTHFD2 cDNA (MTHFD2^R^) restored cell proliferation in *MTHFD2* KO LCLs (Figures 4E and 4F), confirming the role of MTHFD2 in promoting LCL growth and survival.

EBV mimics physiological signals to drive germinal center B cell growth and survival. We hypothesized that 1C metabolism was likewise activated by prototypical agonists operative in germinal center reactions. RNA sequencing (RNA-seq) analysis of resting versus recombinant CD40 ligand (CD40L)-stimulated primary human B cells revealed robust upregulation of mitochondrial 1C enzymes within 24 h post-stimulation that was sustained at 96 h post-stimulation (Figures 4G and 4H). Similarly, stimulation by either B cell receptor cross-linking or Toll-like receptor 9 agonist CpG, but not by interleukin-4 (IL4), induced primary B cell MTHFD2 expression (Figure 4H).

### EBV-Induced Mitochondrial 1C Metabolism Generates Compartment-Specific NADPH

**Figure 5:**
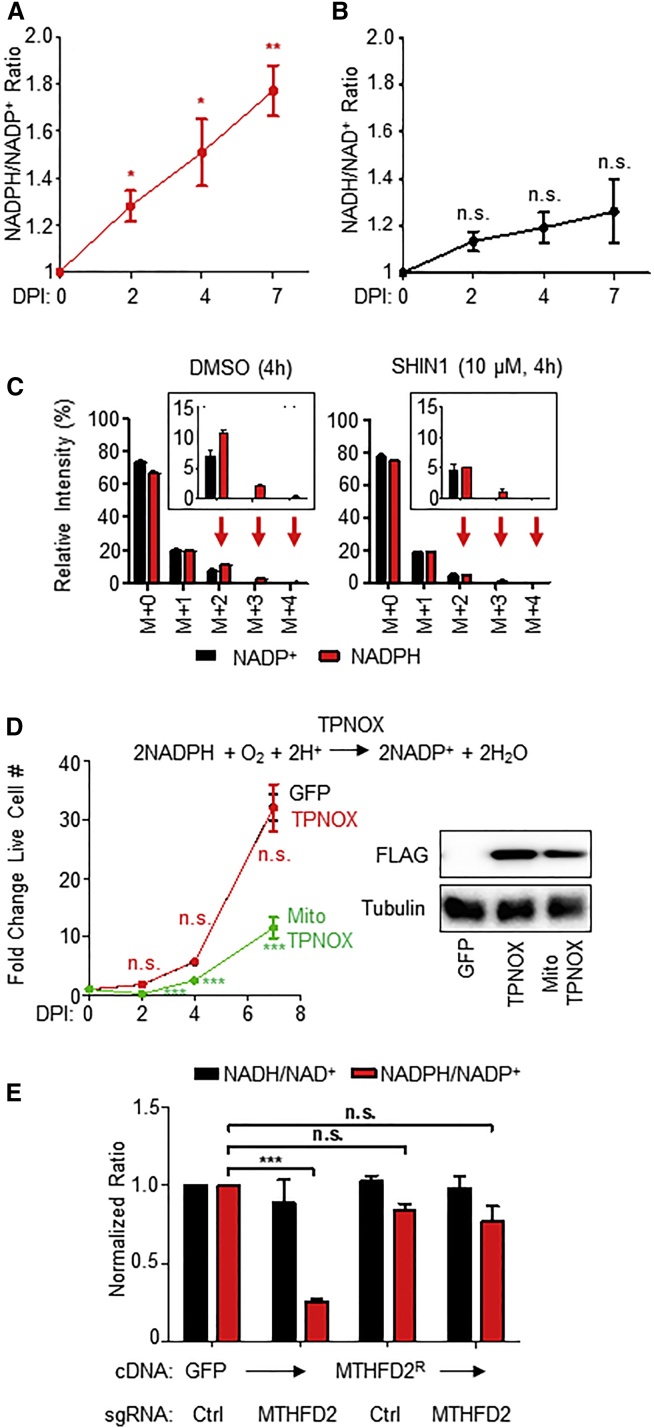
Mitochondrial 1C-derived NADPH Is an EBV Dependency Factor (A) Whole-cell NADPH/NADP^+^ ratios in primary B cells at the indicated time points post-EBV infection. Shown are mean ± SEM values from n = 4 replicates. ^∗^p < 0.05; ^∗∗^p < 0.01 (one-sample t test). (B) Whole-cell NADH/NAD^+^ ratios in primary B cells at the indicated time points post-EBV infection. Data show the mean ± SEM values from n = 4 replicates. n.s., not significant (one-sample t test). (C) LC-MS analysis of NADP^+^ and NADPH cofactors in primary B cells at 4 DPI fed [2,3,3-^2^H]-serine for 4 h in the presence of either DMSO or SHIN1 (10 μM). Data show the mean with SEM, n = 4. Natural isotope correction was not performed. (D) Left: Growth curves of GM12878 LCLs with stable GFP, TPNOX, or MitoTPNOX expression. Data show the mean ± SEM, n = 3. n.s., not significant; ^∗∗∗^p < 0.005 (unpaired two-tailed t test). Right: a representative immunoblot of whole-cell extracts for FLAG-tagged TPNOX or MitoTPNOX and tubulin load-control, n = 3. (E) Quantitation of overall NADH/NAD^+^ (black) and NADPH/NADP^+^ (red) ratios in Cas9+ GM12878 LCLs expressing the indicated control or MTHFD2-targeting sgRNA as well as the indicated *GFP* or *MTHFD2*^*R*^ rescue cDNA. Data show the mean + SEM, n = 3. n.s., not significant; ^∗∗∗^p < 0.005 (one-sample t test). See also Figure S7.

Proteomic profiling highlighted viral induction of anabolic pathways that avidly consume NADPH, including fatty acid and cholesterol biosyntheses (Figure 1D; Table S1). In addition to key roles in providing carbon units for anabolic reactions, mitochondrial 1C metabolism generates reducing power and substrate-level ATP. While the glucose-derived pentose phosphate pathway (PPP) shunt is traditionally considered the major NADPH source, primary B cells do not have robust PPP physiology (Xiao et al., 2018), and EBV downregulated several PPP enzymes, including the rate-limiting enzyme, glucose-6-phosphate dehydrogenase (G6PD) (Table S1). Yet, despite EBV induction of NADPH-consuming pathways, we found that EBV infection significantly increased NADPH/NADP^+^ ratios in newly infected B cells, with little effect on NADH/NAD^+^ ratios out to 7 DPI (Figures 5A and 5B). These results suggest that EBV induces NADPH production, likely through a non-PPP mechanism.

We hypothesized that EBV may utilize mitochondrial 1C as an important NAPDH source. To experimentally determine whether EBV induces NADPH production through mitochondrial 1C pathways, we performed [2,3,3-^2^H]-serine labeling experiments with newly infected cells (Figure 5C). [2,3,3-^2^H]-serine catabolism yields ^2^H-containing 10-formyl-THF, which contributes to deuterium labeling of the adenine backbone of NADP(H) cofactors, as well as redox-active hydrides. Compared to the NADP^+^ labeling pattern, NADPH displayed a shift toward the heavy M+3 and M+4 isotopologues, suggesting that the redox-active hydrogen atoms are derived from exogenous serine through 1C metabolism over the timescale (4 h) of this labeling experiment. 1C blockade by SHIN1 diminished M+3 and M+4 labeling, supporting the idea that 1C metabolism is a crucial means of generating reducing power in the form of NADPH.

To gain insights into compartment-specific NADPH roles in the proliferation of fully transformed LCLs, we utilized the genetically encoded NADPH oxidase triphosphopyridine nucleotide oxidase (TPNOX), which can be expressed as cytosolic or mitochondrial probes (Cracan et al., 2017, Titov et al., 2016). While expression of cytosolic TPNOX had little effect on LCL proliferation, mitochondrial TPNOX significantly diminished LCL growth (Figure 5F), indicating an important intra-mitochondrial NADPH role. In contrast, expression of either TPNOX isoform had little effect on HeLa cell growth (Cracan et al., 2017). Furthermore, the LCL NADPH/NADP^+^ ratio was significantly diminished by MTHFD2 KO and restored by MTHFD2^R^ cDNA rescue (Figure 5G). Conditional EBNA2 inactivation significantly reduced LCL NADPH/NADP^+^ ratio while also inducing a small but significant increase in the NADH/NAD^+^ ratio (Figure S7F), indicating that EBV-induced MTHFD2 has a key role in producing intra-mitochondrial NADPH in support of EBV-transformed B cell growth.

### Serine-Derived Formate Fuels Infected B Cell Nucleotide Synthesis

**Figure 6:**
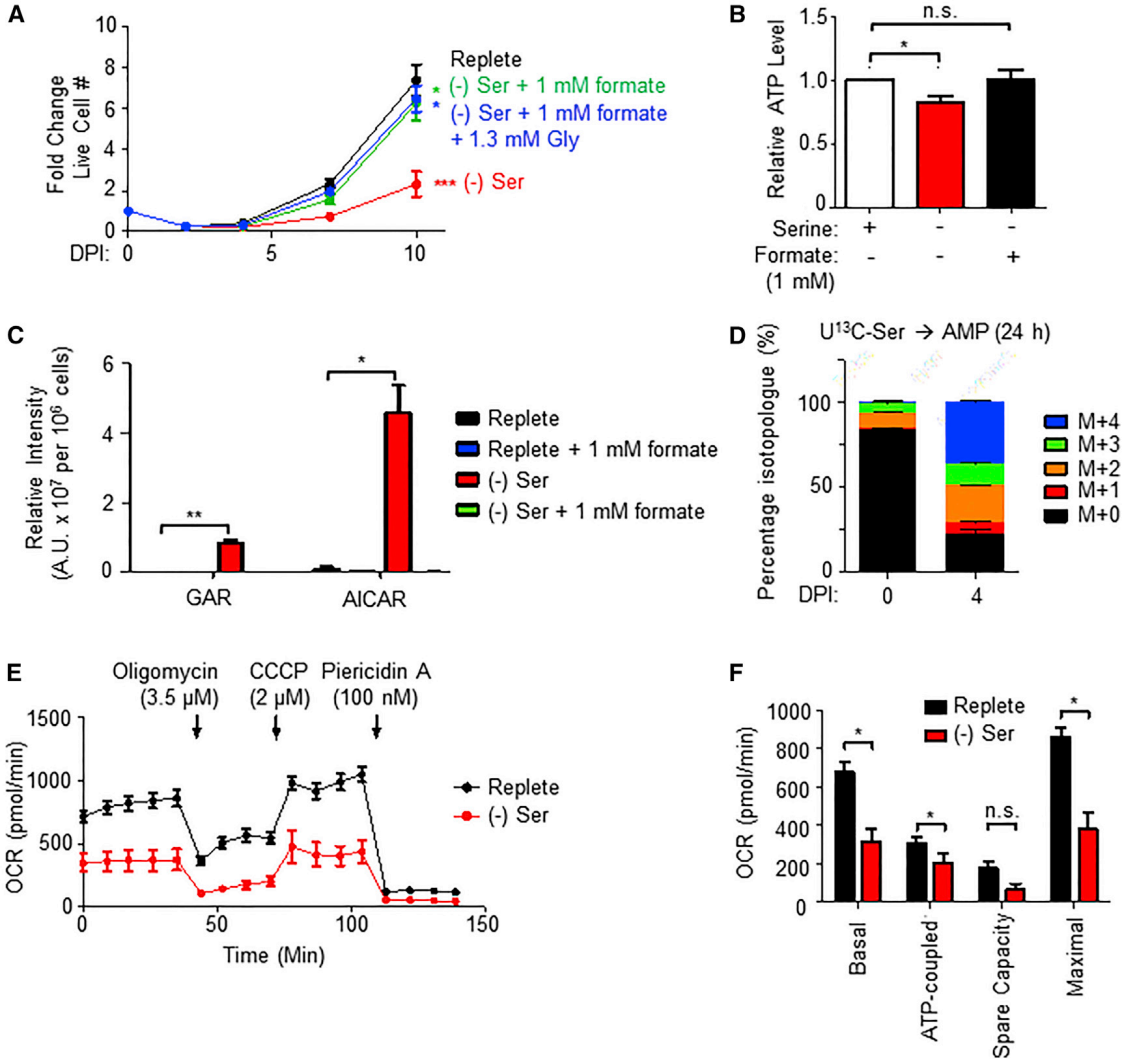
One-Carbon Metabolism Generates Formate for Nucleotide Synthesis (A) Growth curves of newly infected primary B cells cultured in either replete media, serine-deficient media, or serine-deficient media with the indicated supplement (formate ± glycine). Data show the mean ± SEM, n = 7. p < 0.05; ^∗∗∗^p < 0.005 (paired two-tailed t test). (B) Relative ATP levels in primary B cells 7 DPI grown in replete or serine-deficient media, supplemented with formate, as indicated. Data show the mean + SEM, n = 4. n.s., not significant; ^∗^p < 0.05 (one-sample t test). (C) LC-MS detection of *de novo* purine synthesis intermediates GAR and AICAR in newly infected primary B cells 4 DPI. Data show the mean with SEM, n = 3. ^∗^p < 0.05; ^∗∗^p < 0.01 (paired two-tailed t test). (D) LC-MS detection of AMP isotopologues extracted from resting B cells and 4 DPI cells after labeling with U^13^C-serine for 24 h. Data show the mean with SEM, n = 5. (E) Oxygen consumption rates (OCRs) of primary B cells 4 DPI grown in replete or serine-deficient media and subject to flux analysis in the presence of the indicated ETC inhibitors. Data show the mean ± SEM, n = 4. (F) Calculated metabolic parameters of primary cells 4 DPI grown in replete or serine-deficient media. Shown are mean + SEM, n = 4. n.s., not significant; ^∗^p < 0.05 (paired two-tailed t test). See also Figure S7.

EBV triggers B cell transition from quiescence to hyperproliferation, greatly increasing the need for *de novo* nucleotide synthesis. We hypothesized that a major role for EBV-induced 1C metabolism is to provide 1C units and/or glycine for nucleotide synthesis. We therefore tested the extent to which formate supplementation could rescue the outgrowth of EBV-infected cells in serine-deficient media. Exogenous serine withdrawal effects on EBV-driven cell proliferation could be significantly rescued by the 1C donor formate (Figure 6A), suggesting that 1C is a major source of carbon units for nucleotide synthesis in newly infected B cells. Addition of glycine together with formate did not further enhance proliferation (Figure 6A), perhaps due to intrinsic B cell deficiencies in glycine uptake (Ducker et al., 2017). Despite having important roles in nucleotide synthesis, serine withdrawal did not provoke an overt DNA damage response in newly infected cells, as judged by immunoblot for γH2AX (Figure S7G), suggesting that nucleotide-imbalance-related genome instability was not a major cause of growth inhibition. Similarly, serine withdrawal or 1C blockade by SHIN1 did not reduce mitochondrial DNA (mtDNA) or EBV genome copy numbers (Figures S7H–S7K). MTHFD2 KO also did not diminish the mitochondrial membrane potential (Figure S7L). These results suggest that EBV-induced 1C was instead necessary for nucleotide synthesis for other roles, possibly including B cell nuclear genome synthesis and/or RNA transcription.

We hypothesized that serine was required to meet the bioenergetic and biosynthetic requirements of newly infected cell outgrowth. Indeed, exogenous serine withdrawal decreased newly infected B cell ATP levels, which could be reversed with formate supplementation (Figure 6B). Mechanistically, serine deprivation caused the accumulation of *de novo* purine synthesis intermediates glycinamide ribonucleotide (GAR) and 5-aminoimidazole-4-carboxamide ribonucleotide (AICAR), which were completely consumed once cells were supplemented with formate (Figure 6C). Furthermore, U^13^C-serine metabolic tracing readily labeled cellular adenosine monophosphate (AMP) pools, suggesting an important contribution of *de novo* purine biosynthesis in supporting adenine nucleotide levels (Figure 6D). Serine deprivation also diminished newly infected cell basal and maximal OCRs (Figures 6E and 6F). Consistent with an earlier report (Maddocks et al., 2016), serine deprivation did not result in an increase in Thr172 phosphorylation on AMP-activated protein kinase α (AMPKα) (Figure S7G), likely because AMP, ADP, and ATP are equally affected by serine deprivation and the adenylate charge is not substantially altered.

### Viral Activation of Mitochondrial 1C Generates Glutathione for Redox and Glycine Toxicity Defense

Marked upregulation of cholesterol and lipid synthetic pathways during EBV transformation may be necessary for B cell remodeling but likely generates lipid free radicals that can trigger ferroptosis (Yang and Stockwell, 2016). Furthermore, while mitochondrial 1C was recently found to be a key source of B cell lymphoma glycine (Ducker et al., 2017), high 1C flux can generate potentially toxic levels of intracellular glycine, which necessitates disposal through the glycine cleavage system (GCS) or via efflux systems. However, we did not observe significant upregulation of key GCS enzymes such as glycine decarboxylase over the first several DPI at time points of maximal mitochondrial 1C enzyme induction (Table S1). Yet, glycine release into the media was only modestly increased at 4 DPI (Figure S5E). These observations prompted us to investigate if there was a major cellular sink for serine-derived glycine during early infection.

**Figure 7:**
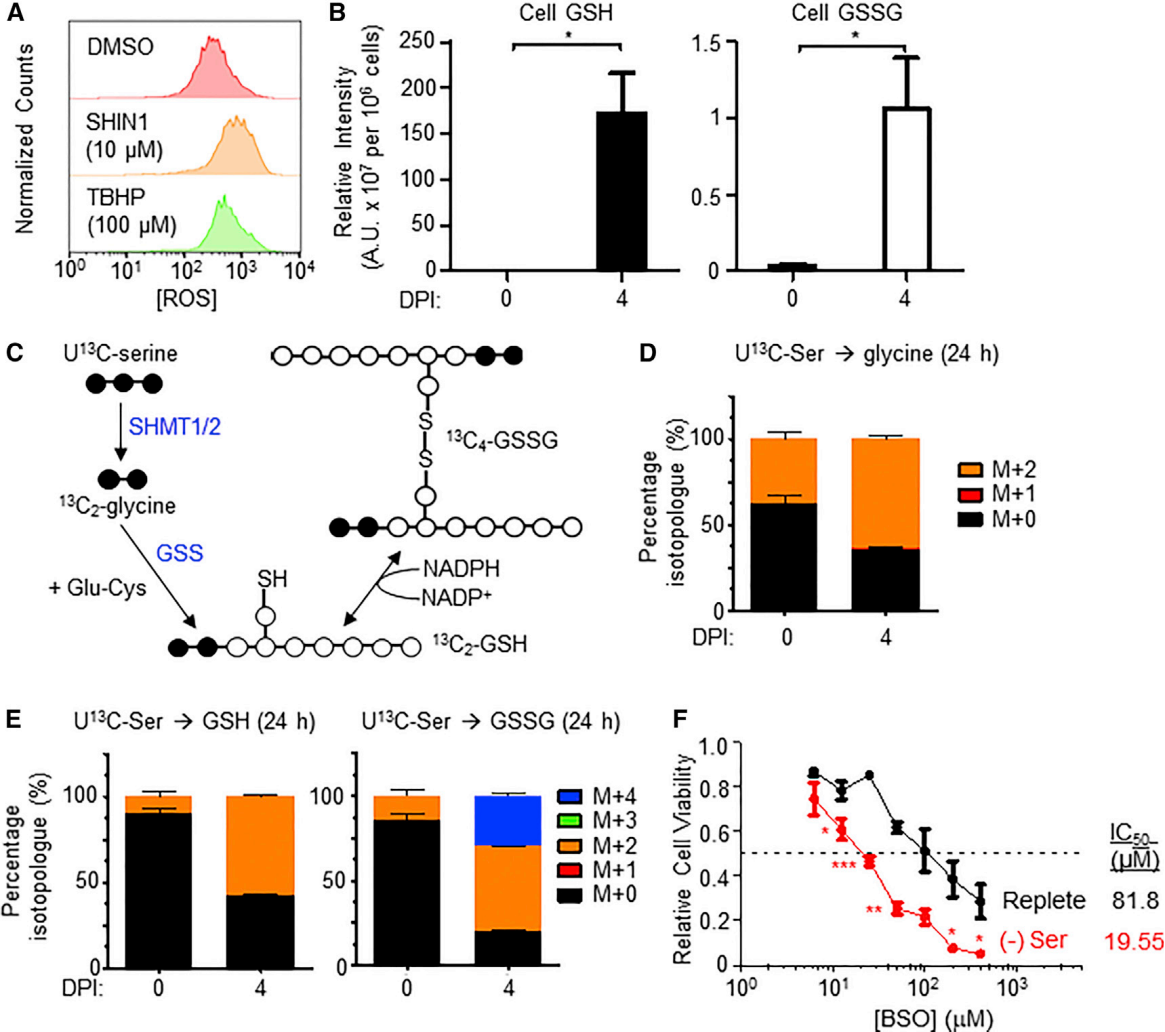
Serine-Derived Glycine Is Used for Glutathione Synthesis and Aids in Redox Homeostasis (A) Flow cytometry of primary B cells 7 DPI treated with either DMSO or SHIN1 (10 μM) and stained with DCFDA for intracellular ROS quantitation. TBHP (100 μM) was used as a positive control. n = 2. (B) LC-MS detection of reduced glutathione (GSH) and oxidized glutathione (GSSG) extracted from primary B cells at 0 and 4 DPI. Data show the mean + SEM, n = 3. ^∗^p < 0.05 (paired one-tailed t test). (C) Schematic showing metabolic tracing of U^13^C-serine-derived glycine in *de novo* synthesis of glutathione. Black circles denote heavy ^13^C atoms, while white circles denote light ^12^C atoms. The reactive thiol bonds are indicated as SH. GSS, glutathione synthase. (D) LC-MS analysis of glycine abundance in metabolite extracts of resting B cells and 4 DPI cells fed U^13^C-serine for 24 h. Data show the mean + SEM, n = 4. (E) LC-MS analysis of GSH and GSSG abundances in metabolite extracts of resting B cells and 4 DPI cells fed U^13^C-serine for 24 h. Data show the mean + SEM, n = 4. (F) Relative viability of primary B cells grown either in replete or serine-deficient media and exposed to the indicated doses of buthionine sulfoximine (BSO) for 3 days from 4 to 7 DPI. Data show the mean ± SEM of n = 3 experiments. ^∗^p < 0.05; ^∗∗^p < 0.01, ^∗∗∗^p < 0.005 (paired one-tailed t test). IC50 values were calculated by non-linear regression analysis. See also Figure S7.

*De novo* glutathione synthesis is central to redox defense and may represent an avenue for glycine disposal without causing overt cytotoxicity. Blockade of the 1C pathway either by SHIN1 (Figure 7A) or MTH-1479 (Figure S7M) increased newly infected B cell intracellular reactive oxygen species (ROS) levels, and EBV infection strongly induced synthesis of glutathione (Figure 7B). We therefore asked if 1C-derived glycine was being utilized for glutathione synthesis. U^13^C-serine tracing labeled approximately 50% of cellular glycine and significant fractions of the intracellular reduced glutathione (GSH) and oxidized glutathione (GSSG) pools (Figures 7C–7E), indicating that serine catabolism was indeed a major source of glycine in the newly infected cell and that 1C-derived glycine was heavily utilized for glutathione synthesis. Further, serine withdrawal significantly increased newly infected B cell sensitivity to buthionine sulfoximine (BSO), which inhibits the first step of glutathione synthesis, as evidenced by the 4-fold decrease in the half maximal inhibitory concentration (IC_50_) levels on cell viability (Figure 7F). These observations are consistent with the ideas that SHMT2 and MTHFD2 are key EBV-induced mediators of NADPH and glutathione production and that a significant role exists for mitochondrial 1C in newly infected cell redox defense. Taken together, these results are consistent with the hypothesis that a second major role for EBV-induced serine catabolism is the provision of metabolites and reducing power for glutathione synthesis to mitigate redox stress and possibly also to serve as a sink for excess glycine produced by 1C metabolism.

## Discussion

Humoral immune responses require B cells to rapidly upregulate metabolic activity in order to support lymphoblast transformation, growth, and survival. This plasticity is necessary for adaptive immune responses, in which B cell clones that successfully recognize foreign antigens are selected and expanded in lymph node germinal center reactions. Through expression of a small number of viral oncoproteins, EBV takes advantage of this plasticity by mimicking germinal center B cell activation signals. Viral subversion of host metabolic pathways underlies EBV’s ability to maintain lifelong carriage and its association with multiple B cell cancers, particularly in immunosuppressed hosts. Yet, a systematic analysis of EBV-mediated metabolic reprograming necessary for B cell activation and transformation has not been performed.

We therefore undertook a near-global scale proteomic analysis of resting primary human B cells and their transition through stages of EBV growth transformation. This approach revealed that EBV targets the mitochondrial 1C pathway, beginning shortly after infection and prior to the first mitosis, a time point where little information has been available. 1C upregulation was a key factor in EBV-induced B cell transformation. Our results provide insights into early events in EBV-mediated metabolic reprograming in B cell growth transformation and have general implications for understanding primary B cell activation.

To support 1C induction and other demands of hyperproliferation, EBV coordinately induced aerobic glycolysis, serine import, and DNSS within the first 4 DPI by means of EBNA2 and its host target MYC. Thus, EBNA2 is a key oncoprotein that mediates adaptation of newly infected B cells into a physiologic state compatible with rapid cell growth. Notably, we also found that BL-like overexpression of MYC was also able to induce mitochondrial 1C. These data suggest that high MYC activity may support germinal-center-dark-zone B cell expansion in humoral responses. EBNA2 also induces EBV oncoproteins, including the CD40 mimic LMP1, which may also serve to support 1C induction in the lymphoblastoid phase time points where EBNA2 and MYC abundances have decreased.

EBV-induced mitochondrial 1C was found to be a major supplier of carbon units for purine, thymidylate, and glutathione synthesis necessary for rapid B cell growth, particularly during BL-like hyperproliferation. Similarly, B cell activation by physiological ligands, including CD40L and B cell receptor stimulation, also upregulated MTHFD2 expression, suggesting similar important roles in physiological B cell humoral responses.

1C metabolism is highly compartmentalized; MTHFD2 ablation was not rescued by reversal of 1C flux through the cytosolic pathway (Scotti et al., 2013), highlighting non-redundancy between the cytosolic and mitochondrial 1C pathways. In contrast to SHMT2 inactivation in primary murine T cells, which could be rescued by formate and the anti-oxidant N-acetyl-cysteine (NAC) (Ron-Harel et al., 2016), we were unable to fully rescue serine deprivation with this combination (data unpublished). Our TPNOX and CRISPR results suggest that generation of intramitochondrial NADPH is a second major role of 1C metabolism in EBV B cell growth transformation. While we cannot exclude that oxygen consumption by mitoTPNOX could have contributed to the observed LCL growth defects, comparable cytosolic TPNOX expression did not cause growth deficit. Likewise, expression of either TPNOX isoform had little effect on HeLa cell growth (Cracan et al., 2017). In contrast to activated primary T cells, serine withdrawal also did not produce overtly deleterious effects on mtDNA replication or cause DNA damage checkpoint activation, suggesting that mitochondrial 1C metabolism may have unique functions in B cell physiology and EBV pathobiology. Additional roles in substrate-level ATP generation remain plausible.

MTHFD2 may boost flux through B cell mitochondrial 1C pathways, as occurs during early embryogenesis (Shin et al., 2014, Shin et al., 2017). Although the isozyme MTHFD2L has key roles in later embryogenesis and in adult tissues, we did not detect its expression in B cells. B cells have been reported to exhibit constitutively low PPP activity, with glucose carbon utilization skewed toward glycolysis (Xiao et al., 2018). Intriguingly, while the phosphatase PP2A was found to redirect glucose carbon units to PPP in DLBCLs, we found PP2A catalytic subunit PPP2CA expression to be unchanged by EBV infection, and EBV suppressed G6PD expression by nearly 2-fold. Furthermore, LCLs can be established from G6PD-deficient human B cell donors (Maeda et al., 1992). Taken together with the finding that MTHFD2 ablation reduced LCL NADPH/NADP^+^ ratios, our results suggest that EBV may induce mitochondrial 1C metabolism in lieu of increasing PPP flux to provide NADPH for B cell transformation, with possible roles in redox defense and anabolic growth. MTHFD2 has been reported to be a major contributor to cellular NADPH in HEK293 cells (Fan et al., 2014) but not previously implicated in primary cell or B cell NADPH generation.

Temporal proteomic analysis revealed that EBV significantly upregulates fatty acid and cholesterol synthesis pathways. Lipid peroxides can be a major source of free radicals that may trigger ferroptosis in the absence of sufficient NADPH and glutathione. Our recent CRISPR analysis of EBV-transformed B cell dependency factors (Ma et al., 2017) found that LCLs are exquisitely dependent on the key glutathione-dependent ferroptosis regulator glutathione peroxidase 4 (GPX4). While resting B cells did not appreciably produce glutathione, EBV infection robustly upregulated serine-dependent glutathione production.

Although EBV robustly induces aerobic glycolysis, we found that an intact ETC was crucial for efficient EBV-induced transformation. While ETC integrity is primarily linked to OXPHOS-dependent ATP generation, interaction with mitochondrial 1C metabolism may also underpin its importance in sustaining proliferative cell growth. Consistent with this hypothesis, SHMT2 inhibition causes impaired mitochondrial translation and defective OXPHOS (Morscher et al., 2018), and ETC dysfunction causes alterations in 1C metabolism, with diminished serine-derived formate production (Bao et al., 2016, Meiser et al., 2016).

Epigenetic silencing plays key roles in EBV B cell growth transformation. Hypermethylation silences host tumor suppressors and viral lytic genes (Saha et al., 2015). Serine-dependent *de novo* ATP synthesis can have a key role in maintaining nucleic acid methylation marks (Maddocks et al., 2016, Stover et al., 2018). It is plausible that newly infected B cells catabolize serine to generate ATP to support methylation of tumor suppressor genes and viral genome CpG islands (Kalla et al., 2010, Kalla et al., 2012). However, under conditions used in this study, where the methyl donors methionine and vitamins B6 and B12 were present in culture, serine is likely not required for DNA and histone methylation. Nonetheless, as a secondary methyl donor, serine levels may become significant *in vivo*, such as in lymphoid germinal center reactions, where localized depletion of primary methyl group donors could occur.

Methotrexate inhibits DHFR and is a cornerstone of antifolate therapies used to target neoplastic and autoimmune B cell conditions. Denis Burkitt and colleagues reported the use of methotrexate as a chemotherapeutic agent to treat endemic BL (Oettgen et al., 1963). Yet, methotrexate and other antifolates in clinical use are limited by toxicities, including effects on rapidly growing gastrointestinal tract epithelia. Folate-dependent mitochondrial 1C metabolism pathway components, specifically MTHFD2, may therefore be attractive targets in the therapeutic treatment of EBV-driven B cell malignancies, given the narrower range of cells that constitutively express this enzyme.

### Limitations of Study

In summary, temporal proteomic profiling provided new insights into metabolic reprograming in EBV-mediated B cell growth transformation and highlighted mitochondrial 1C induction as a key oncogenic event. Our findings underscore mitochondrial 1C metabolism as a critical source of 1C units for purine and thymidylate synthesis, as a means of producing reducing power in the form of NADPH and as a pathway for generating glycine for glutathione synthesis. A key caveat is that we used primary human B cells derived from peripheral blood and infected by EBV *in vitro*. It remains possible that differences in metabolic remodeling exist when EBV infects B cells of distinct differentiation states or in particular *in vivo* niches. We anticipate that the use of additional experimental models, including xenograft, humanized mice, and organoid systems, together with analyses of primary human tumor samples, could provide additional future insights into EBV-driven metabolic remodeling and into the therapeutic potential of 1C pathway blockade. Secondly, although the EBV B95-8 strain efficiently transforms human B cells, a genetic deletion removes certain virus-encoded microRNAs (miRNAs). It is unclear whether these EBV miRNAs may affect 1C metabolism. Thirdly, B95-8 and other type I EBV strains transform B cells more efficiently than type II EBV strains due to sequence differences in EBNA2 (Tzellos et al., 2014). It remains to be determined how polymorphisms in EBNA2 and in other viral genes affect remodeling of B cell metabolism by type II strains.

## STAR★Methods

### Key Resources Table

**Table:** 

REAGENT or RESOURCE	SOURCE	IDENTIFIER
**Antibodies**		

Mouse anti-CD19 APC	BD Biosciences	Cat#555415 RRID:AB_398597
Mouse anti-CD23 PE	BD Biosciences	Cat#555711 RRID:AB_396056
Mouse anti-EBV EBNA1 (OT1x)	Jaap Middledorp	N/A
Mouse anti-EBV EBNA2 (PE2)	Jeffrey Cohen	N/A
Sheep anti-EBV EBNA3A	Exalpha	Cat#F115P RRID:AB_2687621
Sheep anti-EBV EBNA3C	Michelle West and Martin Rowe	N/A
Mouse-anti LMP1 (S12)	Hybridoma	N/A
Rat anti-EBV LMP2A antibody (14B7)	Richard Longnecker	N/A
Mouse anti-EBV gp350 (72A1)	BioXCell	N/A
Mouse anti-γH2AX (Ser139) (JBW301)	Millipore	Cat#05-636 RRID:AB_309864
Rabbit anti-H2AX	Bethyl Laboratories	Cat#A300-083A RRID:AB_203289
Rabbit anti-PHGDH	Bethyl Laboratories	Cat#A304-732A RRID:AB_2620927
Mouse anti-PSAT1	Novus Biologicals	Cat#H00029968-A01 RRID:AB_547380
Rabbit anti-PSPH	Proteintech	Cat#14513-1-AP RRID:AB_2171464
Rabbit anti-SHMT2	Cell Signaling	Cat#12762 RRID:AB_2798018
Rabbit anti-MTHFD2	Proteintech	Cat#12270-1-AP RRID:AB_2147525
Rabbit anti-MTHFD1L	Cell Signaling	Cat#14998 RRID:AB_2798680
Rabbit ant-DDX1	Bethyl Laboratories	Cat#A300-521A RRID:AB_451046
Rabbit anti-c-MYC (N-262)	Santa Cruz	Cat#sc-764 RRID:AB_631276
Rabbit anti-FLAG (DYKDDDDK)	Cell Signaling	Cat#2368S RRID:AB_2217020
Mouse anti-α-tubulin (DM1A)	Abcam	Cat#ab7291 RRID:AB_2241126
Mouse anti-GAPDH (6C5)	Abcam	Cat#ab8245 RRID:AB_2107448
Rabbit anti-ASCT2 (D7C12)	Cell Signaling	Cat#8057 RRID:AB_10891440
Rabbit anti-phospho-AMPKα (Thr172) (40H9)	Cell Signaling	Cat#2535 RRID:AB_331250
Rat anti-ATF4 (W16016A)	Biolegend	Cat#693901 RRID:AB_2650719
Goat anti-rabbit IgG (H+L) Alexa Fluor 488	Invitrogen	Cat#A-11034 RRID:AB_2576217
Goat anti-mouse IgG (H+L) Cross-Adsorbed Secondary Antibody, Alexa Fluor 488	Invitrogen	Cat#A-11001 RRID:AB_2534069
Goat F(ab')_2_ Anti-Human IgM	Southern Biotech	Cat#2022-01 RRID:AB_2795610
Mouse anti-human GLUT1 Alexa Fluor 647 (Clone 202915)	BD Biosciences	Cat#566580

**Bacterial and Virus Strains**		

B95-8		N/A
P3HR-1		N/A

**Chemicals, Peptides, and Recombinant Proteins**		

Tandem mass tag (TMT) 10-plex isobaric reagents	Thermo Fisher	Cat# 90110
HPLC water	VWR	Cat# 23595.328
LC-MS grade acetonitrile	Merck	Cat# 1.00029.2500
Acetonitrile (LC/MS)	Fisher Scientific	Cat#A955-1
Methanol (LC/MS)	Fisher Scientific	Cat#A456-1
Water (LC/MS)	Fisher Scientific	Cat#W6-4
Ammonium acetate (LC/MS)	Sigma	Cat#14267
Ammonium hydroxide (LC/MS)	Fisher	Cat#A470-250
Ammonium carbonate (HPLC)	Fluka	Cat#74415-250G-F
D-Glucose (U-^13^C_6_)	Cambridge Isotope Laboratories	Cat#CLM-1396-1
L-Serine (2,3,3-D_3_)	Cambridge Isotope Laboratories	Cat#DLM-582-0.1
L-Serine (^13^C_3_)	Cambridge Isotope Laboratories	Cat#CLM-1574-H-0.1
Sodium L-lactate (^13^C_3_)	Cambridge Isotope Laboratories	Cat#CLM-1579-0.5
XBridge BEH Amide VanGuard Pre-column, 130Å, 2.5 μm, 2.1 mm X 5 mm, 3/pkg	Waters	Cat#186007763
Xbridge BEH amide 2.5 μm, 2.1 mm X 100 mm	Waters	Cat#186006091
SeQuant® ZIC®-pHILIC (5μm polymer) PEEK 150 x 2.1 mm	EMD Millipore	Cat#150460
Formic acid (LC/MS)	Fisher Scientific	Cat#A117-50
Complete Protease Inhibitor Cocktail	Roche	Cat#11836153001
Magic C4 resin (5 mm, 100 A°)	Michrom Bioresources	Cat#PM5/64100/00
GP118 resin (1.8 mm, 120 A°)	Sepax Technologies	N/A
Piericidin A	Cayman Chemicals	Cat#15379
Tunicamycin, Streptomyces lysosuperficus	Sigma-Aldrich	Cat#654380
Antimycin A from Streptomyces sp.	Sigma-Aldrich	Cat#A8674
8M guanidine hydrochloride solution	Thermo Fisher Scientific	Cat#24115
D-(+)-Galactose	Sigma-Aldrich	Cat#G5388
(Z)-4-Hydroxytamoxifen	Sigma-Aldrich	Cat#H7904
Doxycycline hyclate	Sigma-Aldrich	Cat#D9891
Sodium formate	Fisher Scientific	Cat#S648-500
Glycine	American Bioanalytical	Cat#AB00730-05000
L-Buthionine-sulfoximine	Sigma-Aldrich	Cat#B2515
H2DCFDA (H2-DCF, DCF)	Thermo Fisher Scientific	Cat#D399
2-NBDG (2-(N-(7-Nitrobenz-2-oxa-1,3-diazol-4-yl)Amino)-2-Deoxyglucose)	Thermo Fisher Scientific	Cat#N13195
Propidium iodide - 1.0-mg/mL solution in water	Thermo Fisher Scientific	Cat#P3566
PureLink™RNase A (20 mg/mL)	ThermoFisher Scientific	Cat#12091021
Hoechst 33258, Pentahydrate (bis-Benzimide) - FluoroPure™ Grade	ThermoFisher Scientific	Cat#H21491
Prolong™ Gold Antifade Mountant	ThermoFisher Scientific	Cat#P36930
JC-1 Dye (Mitochondrial Membrane Potential Probe)	ThermoFisher Scientific	Cat#T3168
CellTrace™ CFSE Cell Proliferation Kit	ThermoFisher Scientific	Cat#C34554
7-AAD (7-Aminoactinomycin D)	ThermoFisher Scientific	Cat#A1310
Luperox^®^ TBH70X, *tert*-Butyl hydroperoxide solution	Sigma-Aldrich	Cat#458139
Carbonyl cyanide m-chlorophenylhydrazone (CCCP)	Sigma-Aldrich	Cat#C2759
Carbonyl cyanide 4-(trifluoromethoxy)phenylhydrazone	Sigma-Aldrich	Cat#C2920
Oligomycin A	Sigma-Aldrich	Cat#75351
Cell-Tak Cell and Tissue Adhesive	Corning	Cat#C354240
CBR-5884	Cayman Chemicals	Cat#19236
NCT-503	Cayman Chemicals	Cat#19718
MEGACD40L^®^ Protein (soluble) (human), (recombinant)	Enzo Life Sciences	Cat#ALX-522-110-C010
Recombinant Human IL-4 (carrier-free)	Biolegend	Cat#574004
SHIN1	Raze Therapeutics	
MTH-1479	Raze Therapeutics	

**Critical Commercial Assays**		

CellTiter-Glo® Luminescent Cell Viability Assay	Promega	Cat#G7572
BCA Protein Assay Kit	Thermo Fisher	Cat#23227
Micro BCA Protein Assay Kit	Thermo Fisher	Cat#23235
NAD/NADH-Glo™ Assay	Promega	Cat#G9071
NADP/NADPH-Glo™ Assay	Promega	Cat#G9081
Lactate-Glo™ Assay	Promega	Cat#J5021
Sea horse XF24 FluxPak	Agilent	Cat#100850-001
Seahorse XF24 V7 PET Culture Microplates	Agilent	Cat#101037-004

**Deposited Data**		

Unprocessed peptide files for Figures 1 and S1–S4	This paper	https://data.mendeley.com/datasets/tfg83w73v3/draft?a=be5f697e-07e3-472a-8b1b-736485b18b08
Raw Mass Spectrometry Data Files	This paper	Deposited to the ProteomeXchange Consortium (http://www.proteomexchange.org/) via the PRIDE partner repository with the dataset identifier PRIDE: PXD013034.

**Experimental Models: Cell Lines**		

GM12878	Coriell	N/A
P493-6	Micah Luftig	N/A
2-2-3 EBNA2-HT	Bo Zhao	
P3HR-1 ZHT/RHT	Calderwood et al., 2008	N/A
HEK293T	ATCC	

**Oligonucleotides**		

GCAAAGAGGAGCTGATAGCG	Integrated DNA	PHGDH mRNA Forward
TTCTCAGCTGCGTTGATGAC	Integrated DNA	PHGDH mRNA Reverse
TGGCTGCGACTTCTCTAATGT	Integrated DNA	MTHFD2 mRNA Forward
CCTTCCAGAAATGACAACAGC	Integrated DNA	MTHFD2 mRNA Reverse
CGGCTACCACATCCAAGGAA	Integrated DNA	18S rRNA Forward
GCTGGAATTACCGCGGCT	Integrated DNA	18S rRNA Reverse
GAGCGATCTTGGCAATCTCT	Integrated DNA	BALF5 vDNA Forward
TGGTCATGGATCTGCTAAACC	Integrated DNA	BALF5 vDNA Reverse
ACTTCAACAGCGACACCCACTC	Integrated DNA	GAPDH gDNA Forward
TCTCTTCCTCTTGTGCTCTTGCT	Integrated DNA	GAPDH gDNA Reverse
CACCGCCTCTTACCGAACTGCCGCG	Integrated DNA	MTHFD2 sgRNA1 Forward
AAACCGCGGCAGTTCGGTAAGAGGC	Integrated DNA	MTHFD2 sgRNA1 Reverse
CACCGCCTTCGCCCTTTCCACCTCG	Integrated DNA	MTHFD2 sgRNA2 Forward
AAACCGAGGTGGAAAGGGCGAAGGC	Integrated DNA	MTHFD2 sgRNA2 Reverse
CTTGCAGTGAGCCGAGATT	Integrated DNA	AluYb8 gDNA Forward
GAGACGGAGTCTCGCTCTGTC	Integrated DNA	AluYb8 gDNA Reverse
TGTTGGTTATACCCTTCCCGTACTA	Integrated DNA	MT-ND2 mtDNA Forward
CCTGCAAAGATGGTAGAGTAGATGA	Integrated DNA	MT-ND2 mtDNA Reverse
T∗C∗G∗T∗C∗G∗T∗T∗T∗T∗G∗T∗C∗G∗T∗T∗T∗T∗G∗T∗C∗G∗T∗T (∗=phosphorothioate modification)	Integrated DNA	CpG ODN 2006

**Recombinant DNA**		

pLX_TRC313-MTHFD2^R^	GenScript	N/A
pLX_TRC313-TPNOX	Vamsi Mootha, Massachusetts General Hospital/Howard Hughes Medical Institute, Boston, USA	N/A
pLX_TRC313-MitoTPNOX	Vamsi Mootha, Massachusetts General Hospital/Howard Hughes Medical Institute, Boston, USA	N/A

**Software and Algorithms**		

“MassPike”, a Sequest-based software pipeline for quantitative proteomics.	Steven Gygi Laboratory, Harvard Medical School, Boston, USA	N/A
XLStat	Addinsoft	https://www.xlstat.com/en/
DAVID software	Huang da et al., 2009b	https://david.ncifcrf.gov/
Cluster 3.0	Stanford University University of Tokyo	http://bonsai.hgc.jp/∼mdehoon/software/cluster/software.htm
Java Treeview	SourceForge.net	http://jtreeview.sourceforge.net/
Natural isotope correction	This paper	github.com/BrynMarieR/natural_isotope_correction/

**Other**		

Dialyzed Fetal Bovine Serum	Gemini Bio-Products	Cat#100-108
Standard Fetal Bovine Serum, Qualified, USDA-Approved Regions	ThermoFisher Scientific	Cat#10437028
RPMI 1640 Medium w/o L-Glutamine, L-Serine, HEPES (Powder) - 10L	US Biological	Cat#R8999-15
1X RPMI-1640 Media without Glucose, Glycine and Serine. 500mL, Sterile. 2 Pack.	Teknova	Cat#R9660-02
RPMI 1640 Medium	ThermoFisher Scientific	Cat#11875085
HEPES (1M)	ThermoFisher Scientific	Cat#15630080
RosetteSep™ Human B Cell Enrichment Cocktail	STEMCELL Technologies	Cat#15064
EasySep™ Human B Cell Enrichment Kit	STEMCELL Technologies	Cat#19054
High Precision Glass Cover Slip, box of 100, No 1.5, 24x50mm	Bioscience Tools	Cat#CSHP-No1.5-24x50
Proxeon EASY-nLC 1000 LC pump	ThermoFisher Scientific	LC120
Orbitrap Fusion Lumos Mass Spectrometer	ThermoFisher Scientific	Cat# IQLAAEGAAP FADBMBHQ
LSM 800 with Airyscan	Zeiss	N/A

### Contact for Reagent and Resource Sharing

Further information and requests for resources and reagents may be directed to Benjamin Gewurz (Lead Contact and Corresponding Author; bgewurz@bwh.harvard.edu) or Michael Weekes (Corresponding Author; mpw1001@cam.ac.uk).

### Experimental Model and Subject Details

#### Culture of Established Cell Lines

HEK293T were cultured in DMEM with 10% fetal bovine serum (FBS). GM12878 lymphoblastoid cells were derived from a Caucasian female and were obtained from Coriell. GM12878 Cas9+ cell lines were previously described (Ma et al., 2017). The 2-2-3 EBNA2-HT conditional EBNA2 allele cell line was a kind gift from Bo Zhao and Elliott Kieff (Harvard Medical School) and maintained continuously in the presence of 4-hydroxytamoxifen (4HT). EBNA2-HT cells contain a conditional EBNA2 allele, where EBNA2 is fused to the ligand binding domain of a modified estrogen receptor that binds to 4HT but is not activated by calf estrogens. In the presence of 4HT, EBNA2HT localizes to the nucleus, but upon 4HT withdrawal, it relocalizes to the cytosol and is destabilized. To remove 4HT, cells were washed five times with 4HT-free media with the last two washes 30 minutes each before re-seeding at 300,000 cells per mL. Cells were then grown for a further 48 hours before harvesting for RNA extraction and cell lysate preparation. The P493-6 cell line was a kind gift from Micah Luftig (Duke University). P493-6 cells are LCLs that also contain a conditional EBNA2-HT allele. In addition, they have an exogenous Tet-OFF *MYC* allele, where withdrawal of tetracyclines induces high-level MYC expression. P493-6 cells were maintained continuously in a Burkitt-lymphoma-like state with high exogenous MYC expression by culturing cells in the absence of doxycycline and in the absence of 4HT. To grow P493-6 cells in the lymphoblastoid cell state (which has intermediate MYC level), P493-6 cells were grown in the presence of both 1 μM 4HT to induce EBNA2-HT nuclear translocation and 1 μg/mL doxycycline to suppress exogenous *MYC* allele expression. In this state, EBNA2 induces endogenous MYC expression. To shift P493-6 cells to a low EBNA2 and low MYC state, cells were washed five times and returned to media with 1 μg/mL doxycycline but without 4HT. After 48 hours of growth in any of these conditions, whole cell lysates were prepared. For selection following lentiviral transduction, hygromycin (Calbiochem) at 200 μg/mL or puromycin (Invitrogen) at 3 μg/mL was used. All cells were cultured in RPMI-1640 (Invitrogen) supplemented with 10% standard FBS and penicillin-streptomycin in a humidified incubator at 37°C and at 5% CO_2_. All cells were routinely confirmed to be mycoplasma-negative.

#### Primary Human B-Cell Isolation and Culture

Platelet-depleted venous blood obtained from the Dana-Farber Cancer Institute blood bank were used for primary human B cell isolation, following our Institutional Review Board-approved protocol for discarded and de-identified samples. RosetteSep and EasySep negative isolation kits (STEMCELL Technologies) were used sequentially to isolate CD19+ B-cells with the following modifications made to the manufacturer’s protocols. For RosetteSep, 40 μL of antibody cocktail was added per mL of blood and then layered onto Lymphoprep density medium for centrifugation. For EasySep, 10 μL of antibody cocktail was added per mL of B cells, followed by 15 μL of magnetic bead suspension per mL of B cells. After negative selection, the cells obtained were ≥95% positive for CD19, a nearly pan-B cell surface marker (CD19 is weakly expressed on plasma cells). For most experiments, cells were cultured in RPMI-1640 (Invitrogen) supplemented with 10% standard FBS and penicillin-streptomycin. For metabolite withdrawal and labeling experiments, RPMI-1640 without glucose, serine and glycine (Teknova) was used, and supplemented with 10% dialyzed FBS (Gemini Biosciences) and penicillin-streptomycin and, if applicable, the appropriate chemical supplement. Cells were cultured in a humidified incubator at 37°C and at 5% CO_2_.

### Method Details

#### EBV Infection of Primary B-Cells

EBV B95-8 virus was produced from B95-8 cells with conditional ZTA expression. 4HT was used at a concentration of 1 μM to induce EBV lytic replication, removed 24 hours later, and cells were resuspended in 4HT-free RPMI/10% FBS for 96 hours. Virus-containing supernatants were collected and subject to filtration through a 0.45 μm filter to remove producer cells. Titer was determined experimentally by transformation assay. The P3HR-1 EBV strain was produced by using a P3HR-1 cell line with conditional 4HT-responsive ZTA-HT and RTA-HT alleles, a kind gift from Drs. Eric Johannsen and Elliott Kieff (Ersing et al., 2017, Calderwood et al., 2008). P3HR1 ZHT/RHT cells were induced with 1 μM of 4HT for 24 hours. RPMI/FBS media was then exchanged for fresh media, and viral supernatants were collected from induced cultures 96 hours thereafter. Viral supernatants were purified by filtration through a 0.45 μM filter. Genomic DNA content of preparations of this non-transforming virus were quantitated by PCR for *BALF5* on total DNA extracted, and cross-compared with levels from B95-8 preparation, which were also measured by this approach in a parallel assay at the same time, in order to normalize input virus amounts for subsequent cell infection studies. The plasmid pHAGE-BALF5 was used for standard curves. Calculated genome copy numbers were used to normalize B95-8 and P3HR-1 amounts used for *de novo* infection cross-comparison studies. UV irradiation of B95-8 virus supernatants was performed at a cumulative intensity of 3J per square centimeter on ice, to prevent heat-induced virus degradation. To validate equal B95-8 and P3HR-1 uptake 24 hours post-infection, cells were extensively washed in PBS and then total DNA was extracted from newly infected cells and used for the *BALF5* qPCR assay described above. Immunofluorescence analysis was done for EBNA1 at 48 hours post-infection to further validate equal infection.

#### Agonist Stimulation of Primary B-Cells

Freshly isolated primary B-cells were seeded in complete RPMI media and 10% FBS at 1 million cells per mL. The following agonists were used at these indicated concentrations: MEGACD40L (50 ng/mL), αIgM (1 μg/mL), CpG (1 μM) and IL-4 (20 ng/mL). Cells were harvested at 24 hours and 96 hours for whole cell lysate preparation. For the latter timepoint, agonist replenishment was performed without removal of the spent media at 48 hours.

#### Cell Preparation for Three Biological Replicates of TMT Proteomic Analysis

Using the protocols listed in the Experimental Models section, primary human B-cells were purified by negative selection. For each of three biological replicates, B-cells were isolated from four anonymous human donors. Although we routinely achieved CD19+ B-cell purity >95%, we cultured B-cell preparations from each donor separately, to eliminate the chance of allo-responses from rare co-purifying T-cells between donor cells. With respect to the transformation time course, uninfected B-cells (for the 0 DPI timepoint) were stained with propidium iodide and anti-CD19 antibody, and FACSort was performed for live (based on forward and side scatter parameters) CD19+ B-cells on a BD FACSAria cytometer at the Brigham & Women’s Hospital Flow Cytometry core facility, to control for effects of FACSsort at subsequent timepoints. EBV was added to the remaining purified B-cells at an MOI of 0.1 (approximately 250 μL of supernatant from ZHT cells 5 days after ZHT stimulation, with washout of 4HT after 24 hours, per million purified B-cells). Cells were cultured in a humidified chamber at 37 degrees in complete growth media, again maintaining cultures from each B-cell donor in separate flasks. At each indicated time point, cells from each donor were stained with antibody against CD23, a surrogate marker of EBV-infected cells upregulated by EBNA2 early after EBV infection. Live (based on forward and side scatter gates) CD23+ cells were sorted on the same BD FACSAria cytometer. Whole cell lysates (WCL) and plasma membrane (PM) samples were prepared as described in “*WCL and PM protein preparation for TMT-based proteomics*”. Samples from each donor were sorted sequentially. Immediately following the sort, cells were lysed as described below for WCL analysis or subjected to plasma membrane profiling. Samples were combined at constant ratios at the cell lysis step. The three proteomic time course biological replicates were performed at least one month apart.

#### Protein Preparation for TMT-Based Proteomics

Plasma membrane profiling was performed as described previously (Weekes et al., 2014, Weekes et al., 2012). Briefly, FACS sorted B-cells were washed twice with ice-cold PBS. Sialic acid residues were oxidized with sodium meta-periodate (Thermo Fisher) then biotinylated with aminooxy-biotin (Biotium). The reaction was quenched, cell numbers for each condition were normalized to 2 x 10^6^ using a BioRad TC20 automated cell counter and the biotinylated cells were lysed in 1.6% Triton X-100 lysis buffer. Biotinylated glycoproteins were enriched with high affinity streptavidin agarose beads (Pierce) and washed extensively. Captured protein was denatured with DTT, alkylated with iodoacetamide (IAA, Sigma) and digested on-bead with trypsin (Promega) in 200 mM HEPES pH 8.5 for 3h. Tryptic peptides were collected.

For whole proteome samples, cells were washed twice with PBS, and 150 ul of 6M guanidine/50 mM HEPES pH 8.5 lysis buffer added. Samples were vortexed extensively then sonicated. Cell debris was removed by centrifuging at 13,000 g for 10 min twice. Dithiothreitol (DTT) was added to a final concentration of 5mM and samples were incubated for 20 min. Cysteines were alkylated with 15mM iodoacetamide and incubated 20 min at room temperature in the dark. Excess iodoacetamide was quenched with DTT for 15 min. Samples were diluted with 200 mM HEPES pH 8.5 to 1.5 M guanidine, followed by digestion at room temperature for 3 hr with LysC protease at a 1:100 protease-to protein ratio. Trypsin was then added at a 1:100 protease-to-protein ratio followed by overnight incubation at 37oC. The reaction was quenched with 1% formic acid, samples were spun at 21,000g for 10 min to remove debris and undigested protein, then subjected to C18 solid-phase extraction (Sep-Pak, Waters) and vacuum centrifuged to near-dryness

In preparation for TMT labelling, desalted peptides were dissolved in 200 mM HEPES pH 8.5. For whole proteome samples, peptide concentration was measured by micro BCA (Pierce), and 50 mg of peptide labelled with TMT reagent. For plasma membrane samples, 100% of each peptide sample was labelled. TMT reagents (0.8 mg) were dissolved in 43 μl anhydrous acetonitrile and 5 μl added to peptide sample at a final acetonitrile concentration of 30% (v/v). Samples were labelled as follows: Experiment WCL1: CD19+ uninfected (TMT 126); CD23+ infected d1 (TMT 127N); CD23+ infected d2 (TMT 127C); CD23+ infected d4 (TMT 128N); CD23+ infected d7 (TMT 128C); CD23+ infected d10 (TMT 129N); CD23+ infected d14 (TMT 129C); CD23+ infected d18 (TMT 130N); CD23+ infected d21 (TMT 130C); CD23+ infected d28 (TMT 131). For Experiment WCL2: CD19+ uninfected (TMT 126); CD23+ infected d2 (TMT 127N); CD23+ infected d4 (TMT 127C); CD23+ infected d7 (TMT 128N); CD23+ infected d10 (TMT 128C); CD23+ infected d14 (TMT 129N); CD23+ infected d18 (TMT 129C); CD23+ infected d21 (TMT 130N); CD23+ infected d28 (TMT 130C); CD23+ infected d35 (TMT 131). Experiment WCL3: CD19+ uninfected (TMT 126); CD23+ infected d2 (TMT 127N); CD23+ infected d4 (TMT 127C); CD23+ infected d7 (TMT 128N); CD23+ infected d10 (TMT 128C); CD23+ infected d14 (TMT 129N); CD23+ infected d18 (TMT 129C); CD23+ infected d21 (TMT 130N); CD23+ infected d24 (TMT 130C); CD23+ infected d28 (TMT 131). Experiment PM1: CD19+ uninfected (TMT 126); CD23+ infected d2 (TMT 127N); CD23+ infected d4 (TMT 127C); CD23+ infected d7 (TMT 128N); CD23+ infected d10 (TMT 128C); CD23+ infected d14 (TMT 129N).

Following incubation at room temperature for 1 hr, the reaction was quenched with hydroxylamine to a final concentration of 0.5% (v/v). TMT-labeled samples were combined at a 1:1:1:1:1:1:1:1:1:1 ratio. The sample was vacuum-centrifuged to near dryness and subjected to C18 solid-phase extraction (SPE) (Sep-Pak, Waters).

Offline high pH reversed-phase fractionation of peptides from experiments WCL1-3, and offline tip-based strong cation exchange fractionation of the PM sample were performed, and WCL peptide fractions combined as described previously (Weekes et al., 2014).

#### Peptide Fragmentation and Detection by LC-MS3

Mass spectrometry data were acquired using an Orbitrap Lumos coupled with a Proxeon EASY-nLC 1000 LC pump (Thermo Fisher Scientific, San Jose, CA). Peptides were separated on a 75 mm inner diameter microcapillary column packed with 0.5 cm of Magic C4 resin (5 mm, 100 A°, Michrom Bioresources) followed by approximately 20 cm of GP118 resin (1.8 mm, 120 A°, Sepax Technologies). Peptides were separated using a 3 hr gradient of 6 to 30% acetonitrile in 0.125% formic acid at a flow rate of 300 nl/min. Each analysis used an MS3-based TMT method (McAlister et al., 2012, Ting et al., 2011). The scan sequence began with an MS1 spectrum (Orbitrap analysis, resolution 120,000, 350-1400 Th, AGC target 5 x 10^5^, maximum injection time 100 ms). ‘Rapid’ was selected for MS2 analysis, which consisted of CID (quadrupole ion trap analysis, AGC 1.8 x 10^4^, NCE 35, maximum injection time 120 ms). For MS3 analysis, precursors were fragmented by HCD prior to Orbitrap analysis (NCE 55, max AGC 2 x 10^5^, maximum injection time 150 ms, isolation specificity 0.7 Th, resolution 50,000).

#### Analysis of LC-MS3 Data

Mass spectra were processed using a Sequest-based in-house software pipeline as described previously (Weekes et al., 2014). Briefly, MS spectra were converted to mzXML using a modified version of ReAdW.exe. A combined database was constructed from (a) the human Uniprot database (February 4^th^, 2014), (b) B95-8 strain EBV (see ‘Database Generation’ section), (c) all open reading frames from a six-frame translation of B95-8 strain EBV and (d) common contaminants such as porcine trypsin and endoproteinase LysC. The combined database was concatenated with a reverse database composed of all protein sequences in reversed order. Searches were performed using a 20 ppm precursor ion tolerance. Product ion tolerance was set to 0.03 Th. TMT tags on lysine residues and peptide N termini (229.162932 Da) and carbamidomethylation of cysteine residues (57.02146 Da) were set as static modifications, while oxidation of methionine residues (15.99492 Da) was set as a variable modification. To control the fraction of erroneous protein identifications, we used a target-decoy strategy (Elias and Gygi, 2007, Elias and Gygi, 2010). Peptide spectral matches (PSMs) were filtered to an initial peptide-level false discovery rate (FDR) of 1% with subsequent filtering to attain a final protein-level FDR of 1%. PSM filtering was performed using a linear discriminant analysis, as described previously (Huttlin et al., 2010), considering the following parameters: XCorr, DCn, missed cleavages, peptide length, charge state, and precursor mass accuracy. Protein assembly was guided by principles of parsimony to produce the smallest set of proteins necessary to account for all observed peptides. Data for all three biological WCL replicates and the PM analysis were initially filtered and assembled together to produce a single list of quantified proteins.

Proteins were quantified by summing TMT reporter ion counts across all matching peptide-spectral matches using in-house software, as described previously (Pease et al., 2013). Briefly, a 0.003 Th window around the theoretical m/z of each reporter ion (126, 127N, 127C, 128N, 128C, 129N, 129C, 130N, 130C, 131) was scanned for ions, and the maximum intensity nearest to the theoretical m/z was used. We required every individual peptide used for quantitation to contribute sufficient TMT reporter ions (minimum of 1,250 per spectrum) so that each on its own provided a representative picture of relative protein abundance (McAlister et al., 2012). We additionally employed an isolation specificity filter to minimize peptide coisolation (Ting et al., 2011). Peptide-spectral matches with poor quality MS3 spectra (more than 9 TMT channels missing and/or a combined signal:noise ratio of less than 250 across all TMT reporter ions) or no MS3 spectra at all were excluded from quantitation. Protein quantitation values were exported for further analysis in Excel. For protein quantitation, reverse and contaminant proteins were removed, then each reporter ion channel was summed across all quantified proteins and normalized assuming equal protein loading across all 10 samples. Gene Ontology terms were downloaded from www.Uniprot.org. XLStat (Addinsoft) was used to determine the number of distinct k-means clusters (Figure S1D). Hierarchical centroid clustering was based on Euclidian Distance. Hierarchical and k-means clustering were performed using Cluster 3.0 (Stanford University) and visualized using Java Treeview (http://jtreeview.sourceforge.net).

Pathway Analysis was performed using the Database for Annotation, Visualization and Integrated Discovery (DAVID) (Huang da et al., 2009b) version 6.8 with default settings. A given cluster was always searched against a background of all proteins quantified within the relevant experiment. To generate lists of proteins for DAVID enrichment analysis, for WCL data, proteins were included if they were quantified in all three experiments WCL1-3. For Figure 1D, parent terms were either derived from a hierarchical structure in Uniprot, or Gene Ontology (http://supfam.org/SUPERFAMILY/cgi-bin/go.cgi).

##### Gene Set Enrichment Analysis (GSEA)

The difference in the proteomics data from uninfected cells was used to generate a ranked list for GSEA Preranked analysis using the Molecular Signatures Database v5.2 (C7:immunologic signatures) (Subramanian et al., 2005). Gene sets with nominal p value < 0.05 and false discovery rate (FDR) <0.25 were defined as significantly enriched gene sets, which were selected for visualization.

#### LC-MS Metabolite Analysis

Cells were seeded at 1 million cells/mL with fresh media (containing labeled amino acids, if required) 24 hours prior to harvesting. To prepare cellular metabolite extracts, cells were washed once with ice-cold PBS and once with ice-cold 100 mM ammonium acetate before lysis with acetonitrile-methanol-water (27:9:1). For detection of cellular M+3 serine with U^13^C-glucose, the same procedure was used with the labeling period shortened to 4 hours to minimize exchange between cellular serine and media serine. For nicotinamide dinucleotide hydride labeling, we followed the protocol described by Fan et al. (2014) and Zhang et al. (2017), which did not involve natural isotope correction. Briefly, 1 million cells were labeled with serine-free media supplemented by 285 μM [2,3,3-^2^H]-serine for 4 hours, and cell pellets were lysed directly in 200 μL ice-cold acetonitrile-methanol-water (27:9:1) containing 0.1 M formic acid. Wash steps were omitted to avoid hydride exchange. Lysates were immediately neutralized with 17.5 μL of 15% (w/v) ammonium bicarbonate solution (within 15 seconds of lysis buffer addition) and centrifuged at maximum speed for 20 min at 4°C. Supernatants were analyzed on the same day by the ZIC-pHILIC method mentioned below. To prepare media metabolite extracts, 50 μL of media was extracted with acetonitrile-methanol (3:1). All extraction steps were performed at 4°C. Crude extracts were centrifuged at maximum speed at 4°C for 20 minutes to remove cellular debris, and supernatants were either used immediately or frozen at -80°C for short-term storage. LC/MS-based analyses were performed on a Q Exactive Plus orbitrap mass spectrometer equipped with an Ion Max source and a HESI II probe, which was coupled to a Dionex UltiMate 3000 UPLC system (Thermo Fisher Scientific).

Polar metabolites in the spent media and cellular extracts were analyzed using Xbridge BEH Amide XP HILIC 2.5 μm, 2.1 mm x 100 mm column (Waters) with the guard column and SeQuant ZIC-pHILIC Polymeric 5 μm, 150 x 2.1 mm column (EMD-Millipore).

For the amide method, the mobile phase A was 5% acetonitrile, 20mM ammonium acetate/ammonium hydroxide, pH 9. The mobile phase B was 100% acetonitrile. The flow rate was 220 μl/min from 0 – 15 min, and 420 μl/min from 15 – 25 min. The gradient was as follows: 0 min: 85% B; 0.5 min: 85% B; 9 min: 35% B; 11 min: 2% B; 13.5 min: 85% B; 15 min: 85% B; 22 min: 85% B. The MS data was collected in the polarity switching mode with full scan mode in a range of 70–1000 m/z, with the resolution at 70,000, the AGC target at 1E6, and the maximum injection time at 80 ms, the sheath gas flow at 50 units, the auxiliary gas flow at 10 units, the sweep gas flow at 2 units, the spray voltage at 2.5 kV, the capillary temperature at 310°C, and the auxiliary gas heater temperature at 370°C.

For dTTP, AMP, GSH and GSSG using the ZIC-pHILIC method, the mobile phase A was 20mM ammonium carbonate/ammonium hydroxide, pH 9.6. The mobile phase B was 100% acetonitrile. The flow rate was 150 μl/min. The gradient was as follows: 0 min: 80% B; 0.5 min: 80% B; 20.5 min: 20% B; 21.5 min: 80% B; 29 min: 80% B. The MS data acquisition was collected in the polarity switching mode with full scan mode in a range of 70–1000 m/z, with the resolution at 70,000, the AGC target at 1E6, and the maximum injection time at 80 ms with the same parameters mentioned above.

For NAD(P)^+^ and NAD(P)H using the ZIC-pHILIC method, the mobile phase A was 20mM ammonium carbonate/ammonium hydroxide, pH 9.6. The mobile phase B was 100% acetonitrile. The flow rate was 110 μl/min. The gradient was as follows: 0 min: 80% B; 2 min: 80% B; 18 min: 62% B; 25 min: 20% B; 26 min: 80% B; 37 min: 80% B. The MS data was collected in the negative polarity mode with full scan mode in a range of 300–1000 m/z, with the resolution at 70,000, the AGC target at 3E6, and the maximum injection time at 400 ms, and the same parameters mentioned above.

Progenesis QI software (Waters, NC) and Xcalibur (Thermo Fisher Scientific) were used to analyze the data. Absolute quantification of glucose, serine and lactate in the spent media was measured by comparing to the 13C-labeled internal standards at different spiked concentrations.

#### Natural Isotope Correction

Mass isotopologue distributions were corrected for the natural abundance of heavy isotopes of hydrogen, carbon, oxygen, and nitrogen. Phosphorus is considered monoisotopic. Natural abundances were found in the Table of Isotopic Compositions of the Elements (TICE) maintained by the International Union of Pure and Applied Chemistry (IUPAC) (Meija et al., 2016).

**Table:** 

Element	M+0	M+1	M+2
H	0.999885	0.000115	
C	0.9893	0.0107	
O	0.99757	0.00038	0.00205
N	0.99632	0.00368	
P	1		

This method takes into account the different isotope abundance distributions in the labeled molecule as compared to the isotope abundance distributions in the unlabeled molecule (the proportionality “skew”). The corrected mass isotopologue distribution MID_corr_ is obtained by multiplying the observed mass isotopologue distribution (MID_obs_) by a correction matrix (CM); the CM for dTTP is shown below:

(1) $$\begin{array}{cc} \left[\begin{array}{c} M0_{obs}\\ M1_{obs}\\ M2_{obs}\\ \vdots \end{array}\right] & =CM_{dTTP}\left[\begin{array}{c} M0_{corr}\\ M1_{corr}\\ M2_{corr}\\ \vdots \end{array}\right] \end{array}$$

(2) $$\begin{array}{ccc} & = & CM_{H_{17}}CM_{C_{10}}CM_{O_{14}}CM_{N_{2}}CM_{P_{3}}\left[\begin{array}{c} M0_{corr}\\ M1_{corr}\\ M2_{corr}\\ \vdots \end{array}\right] \end{array}$$

Using the example of the oxygen correction matrix, a correction matrix for any non-labelled atom type takes the form:

CMO14=[p(O1416)000…p(O1316·O117)p(O1416)00…p(O1216·O217)+p(O1216·O181)p(O1316·O117)p(O1416)0…⋮⋮⋮⋮⋱]

Defining:

$$\begin{array}{ll} p\left(\text{elemental}\;\text{isotopomer}\right) & =\left(\begin{array}{c} N\\ f\left(I_{i}\right),\ldots ,f\left(I_{n}\right) \end{array}\right)\times \prod_{i=1}^{n}p{\left(I_{i}\right)}^{f\left(I_{i}\right)}\\ & =N!\times \prod_{i=1}^{n}\left(\frac{p{\left(I_{i}\right)}^{f\left(I_{i}\right)}}{f\left(I_{i}\right)!}\right) \end{array}$$

Where *N* is the number of atoms of the element (in this case, oxygen) in the molecule, *n* is the number of naturally occurring isotopes, I_1_, … *I*_*n*_, of the element, p(I_*i*_) gives the natural abundance of isotope *I*_*i*_, and f(I_*i*_) is the frequency of the *i*-th isotope in the molecule.

The correction matrix for hydrogen takes a special form since hydrogen is the element being labelled:

CMH17=[p(H171)000……p(H161·H12)p(H161)00……p(H151·H22)p(H161·H12)p(H151)0……⋮⋮⋮⋮⋱p(H172)p(H162)p(H152)……1]

We used a least-squares implementation of the accepted optimal approach for natural abundance correction, the “skewed” method as outlined by Midani et al. in reference to earlier works by Rosenblatt and Fernandez et al. (Midani et al., 2017, Fernandez et al., 1996, Rosenblatt et al., 1992). The R package *nnls* was used to find the non-negative least squares solution. Details of algorithmic implementation were adapted from Yang et al. (Yang et al., 2009). Full code is available online at github.com/BrynMarieR/natural_isotope_correction/.

#### Flow Cytometry Analysis

Flow cytometry studies (FACS) were performed on a BD FACSCalibur instrument. Cells were washed once with cold PBS supplemented with 0.5% bovine serum albumin (BSA). Cells were then incubated with a 1:100 dilution of fluorophore-conjugated primary antibody (and, if applicable, a 1:500 dilution of fluorophore-conjugated secondary antibody) in PBS with 0.5% BSA for 1 hour. Cells were pelleted and resuspended in 400 μL of PBS, strained into flow cytometry-compatible tubes and processed immediately with a flow cytometer. Sample processing was performed either immediately or within 24 hours after staining.

For DCFDA staining, cells were treated with 10 μM DCFDA in complete media for 20 minutes before processing with the flow cytometer. As a positive control, pre-treatment of cells with 100 μM tert-butyl hydrogen peroxide (TBHP) for 3 hours was performed. For JC-1 staining, cells were treated with 10 μM JC-1 in complete media for 15 minutes before processing with the flow cytometer. As a positive control, concurrent treatment of cells with 100 μM CCCP was performed. For 2-NBDG staining, cells were incubated with 10 μg/mL 2-NBDG in complete media for one hour at 37°C. For CFSE staining, 4 DPI cells and GM12878 LCLs were stained with 10 μM CFSE for 15 minutes at 37°C, washed and resuspended at 100 000 cells/mL. For 7-AAD staining, cells were stained with 1 μM 7-AAD at room temperature for 5 minutes before being placed at ice until further processing with the flow cytometer. For propidium iodide staining, 7DPI cells and GM12878 LCL fixed in ice-cold 90% ethanol/PBS for at least two days were re-hydrated in PBS and stained with a solution of 20 μg/mL propidium iodide, 40 μg/mL RNase A and 1:1000 (v/v) Triton X-100 for 30 minutes before immediate processing by the flow cytometer.

Flow cytometric data was acquired with a BD FACSCalibur instrument in most instances (with the exception of the CFSE and 7-AAD experiments, whose data were acquired with a BD FACSCanto I flow cytometer) and analysis was performed with FlowJo.

#### Immunofluorescence Microscopy

Approximately 1 million cells were pelleted and resuspended in 1-2 μL PBS and streaked onto glass slides to dry. Cells were fixed with 4% paraformaldehyde/PBS solution for 10 minutes and permeabilized with 0.5% (v/v) Triton X-100/PBS solution for 5 minutes, with PBS washing in between and after each of those steps. Cells were incubated with 20% normal goat serum (NGS) blocking reagent for 1 hour. EBNA1-specific OT1x antibody was diluted 1:100 in the blocking reagent and added to cells for 1 hour. Cells were washed and incubated with Alexa Fluor 488-conjugated goat anti-mouse secondary antibody (diluted 1:1000 in blocking reagent) for 30 minutes. Cells were washed and incubated with a 10 μg/mL Hoechst 33258/PBS solution for 3-5 minutes. Cells were washed and successively dehydrated in 70% (1 minute), 90% (1 minute) and 100% ethanol (1 minute). ProLong Gold antifade reagent was added to each well and a No. 1.5 coverslip was attached. Data acquisition was performed using a Zeiss LSM 800 instrument. Processing and analysis were performed using ZEN Blue software (Zeiss). All steps were performed at room temperature.

#### *In Vitro* Transformation Assays

Freshly isolated primary human B-cells, purified as outlined above by negative selection, were seeded into 96-well plates at a density of 500,000 cells/mL in 100 μL per well of RPMI/10% FBS. B95-8 virus supernatant (see section entitled, “*EBV infection of primary B-cells*”, for details) was diluted ten-fold to give a five-point dilution series. To each well, 100 μL of virus supernatant was added. DMSO vehicle, MTH-1479 or SHIN1 were added at the indicated concentrations (5 or 10 μM). Vehicle or drug were refreshed every 3 to 4 days by carefully aspirating 100 μL of spent media and replenishing with fresh drug-containing media. At four weeks post-infection, the proportion of wells with B-cell outgrowth was plotted against the dilution of virus supernatant used per well, as previously described (Henderson et al., 1977). One transforming unit per well was defined as the amount of virus required to attain B-cell outgrowth in 62.5% of wells.

#### Growth Curve Analysis

Newly infected B-cells were seeded at 500,000 cells per well in a volume of 1 mL of RPMI/FBS and grown for two days before measurements of cell numbers were made. For GM12878 lymphoblastoid cells, 100,000 cells were seeded per well in a volume of 1mL and grown for two days prior to measurements of live cell number. For live cell measurements, cells were pelleted and resuspended in the same volume of media, trypan blue was added, and live cell number was quantitated with the TC20 automatic cell counter (Bio-Rad). After measurements were taken, cells were passaged accordingly to give 100,000 cells per mL in RPMI/10% FBS and grown for two more days. The same procedure was repeated to obtain measurements at later time points. At each time point, cells were treated with the appropriate inhibitor and/or rescue metabolite(s) at the indicated concentrations after passaging.

#### CRISPR Editing in GM12878 LCL

Single guide RNA (sgRNA) constructs were generated as previously described (Jiang et al., 2018) using sgRNA sequences from the Broad Institute Avana Library. The MTHFD2^R^ cDNA rescue construct utilizes the pLX_TRC313 (Hyg^R^) vector backbone and was synthesized and sequence-validated by GenScript. C166T and G349A silent point mutations were introduced into the MTHFD2 cDNA sequence to produce synonymous mutations that conferred resistance to sgRNA #1 and sgRNA #2, respectively.

LCL CRISPR editing was performed as previously described (Jiang et al., 2018). Briefly, lentiviruses encoding sgRNAs were generated by transient transfection of 293T cells with packaging plasmids and pLentiGuide-Puro plasmids. GM12878 cells stably expressing Cas9 were transduced with the lentiviruses and selected with 3 μg/mL puromycin for three days before replacement with antibiotic-free media. For rescue experiments or cDNA overexpression, 293T cells were transiently transfected to produce lentiviruses that carry the rescue or control GFP cDNA and a hygromycin resistance marker. GM12878 cells were transduced with 293T rescue lentivirus supernatants at 48 and 72 hours post-293T cell transfection, and selected into RPMI/10% FBS with 200 μg/mL hygromycin 48 hours later. Cells were hygromycin selected for at least one week before transduction with sgRNA-encoding lentiviruses. CRISPR editing and rescue cDNA expression were confirmed by immunoblot.

#### Kit-Based Quantitation of ATP, NAD(P)H and Extracellular Lactate

Cells were assayed using commercially available kits (Promega), namely CellTiter-Glo (ATP measurements), NADH-Glo (NADH and NAD^+^ measurements), NADPH-Glo (NADPH and NADP^+^ measurements) and Lactate-Glo, accordingly to manufacturer’s instructions.

#### Quantitative PCR

RT-qPCR analysis of mRNA abundance was performed on a BioRad CFX Connect Real-time PCR detection system, using Power SYBR Green RNA-to-CT 1-Step Kit (Applied Biosystems) for 40 cycles. Expression values relative to 18S rRNA expression were calculated using CFX Manager Software. Quantitative PCR of viral genome copies utilized host cell *GAPDH* gene copy number as control. To quantitate mitochondrial DNA copy number, total cellular DNA was extracted using the DNAeasy kit (Qiagen). Quantitative PCR was then performed on 10 ng input DNA using *MT-ND2* (mtDNA)-specific and *AluYb8* (nuclear DNA)-specific primers. C_t_ values obtained from *AluYb8* amplification were used for normalization. A list of PCR primers used can be found in the Key Resources Table.

#### Mitochondrial Stress Test

Cell culture plates were layered with Cell-Tak to enable adhesion of the B-cells. Cells were seeded at 500,000 per well. For standard measurements, complete bicarbonate-free RPMI-1640 supplemented with 25 mM HEPES, 10% dialyzed FBS and 2 mM L-glutamine was used as the growth media during the period of data acquisition. For serine withdrawal and subsequent rescue experiments, complete bicarbonate- and serine-free RPMI-1640 supplemented with 25 mM HEPES, 10% dialyzed FBS and 2 mM L-glutamine was used, with serine and formate added accordingly to the appropriate wells. Detection of changes in oxygen consumption and extracellular acidification rates was achieved with the use of Seahorse XF24 sensor cartridges. The following mitochondrial poisons were used: 3.5 μM oligomycin, 2 μM CCCP and 100 nM piericidin A. Data acquisition was performed with the Seahorse XF24 Extracellular Flux Analyzer (Agilent).

### Quantification and Statistical Analysis

The exact value of replicates (n) is indicated in the figure legends and refers to the number of biological replicates. Blinding or sample-size estimation was not appropriate for this study. There were no inclusion criteria and no data was excluded. Unless stated otherwise, Student’s t-test was performed for all experiments. GraphPad Prism was used to plot data and to perform subsequent statistical analysis. FlowJo was used to process and analyze flow cytometry data.

### Data and Software Availability

Unprocessed peptide data files for Figures 1 and S1–S4 are available at https://data.mendeley.com/datasets/tfg83w73v3/draft?a=be5f697e-07e3-472a-8b1b-736485b18b08. These files include details of peptide sequence, redundancy, protein assignment raw unprocessed TMT reporter intensities and isolation specificity. The mass spectrometry proteomics data have been deposited to the ProteomeXchange Consortium (http://www.proteomexchange.org/) via the PRIDE partner repository. The accession number for the mass spectrometry proteomics data reported in this paper is PRIDE :PXD013034.

## Supplemental Information

Document S1. Figures S1–S7

Table S1. Interactive Plotter and Summary Table for All Proteomic Data from Experiments WCL1-3 and PM1, Related to Figure 1 The “Plots” worksheet generates graphs for all the human and viral proteins quantified. The “Data” worksheet shows minimally annotated protein data, for which the only modifications are formatting and normalization.

Table S2. Full Details of Enriched Terms from Figure 1D, Related to Figure 1

Table S3. Full Details of Enriched Terms from Figure 2A, Related to Figure 2

Document S2. Article plus Supplemental Information
