# Supplementary material for: Epstein-Barr-Virus-Induced One-Carbon Metabolism Drives B Cell Transformation
Source: Cell Metab. 2019 Sep 3;30(3):539–555.e11. doi: 10.1016/j.cmet.2019.06.003 (PMC6720460; doi:10.1016/j.cmet.2019.06.003)
Supplement: Document S1. Figures S1–S7 [file mmc1.pdf]

Cell Metabolism, Volume *30*

Supplemental Information

Epstein-Barr-Virus-Induced One-Carbon Metabolism Drives B Cell Transformation

Liang Wei Wang, Hongying Shen, Luis Nobre, Ina Ersing, Joao A. Paulo, Stephen Trudeau, Zhonghao Wang, Nicholas A. Smith, Yijie Ma, Bryn Reinstadler, Jason Nomburg, Thomas Sommermann, Ellen Cahir-McFarland, Steven P. Gygi, Vamsi K. Mootha, Michael P. Weekes, and Benjamin E. Gewurz

Figure S1

A

	All peptides	Human proteins	EBV proteins
**WCL1**	73146	7627	21
**WCL2**	52270	6986	17
**WCL3**	62810	7255	22
**All3 WCL**		6445	11
**PM1**	3988	712	2

B

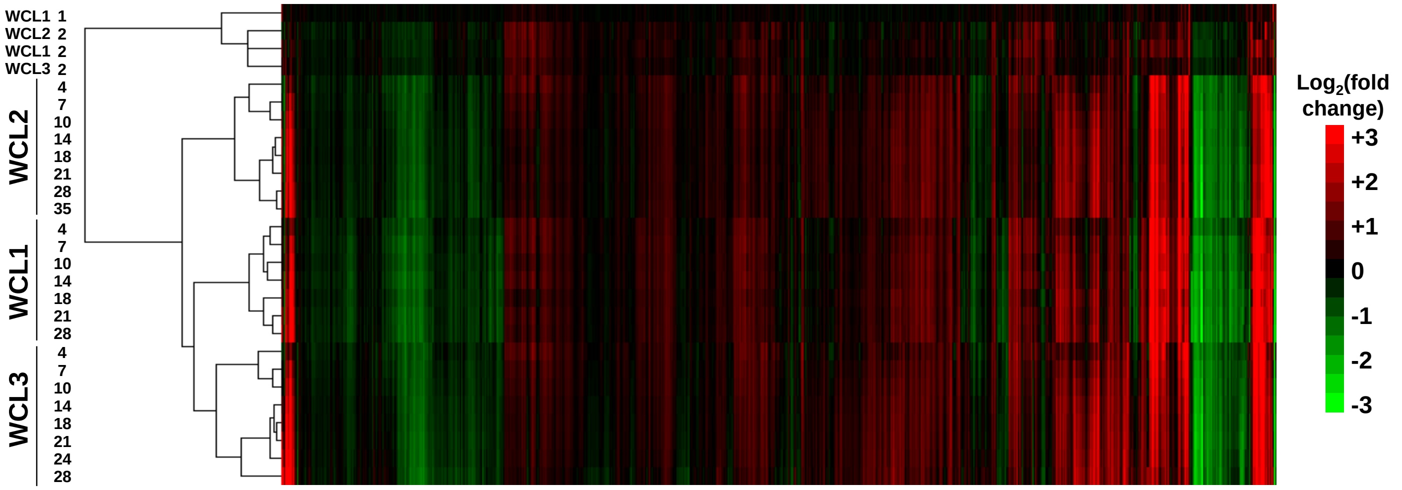

C

	WCL1 1	WCL1 2	WCL1 4	WCL1 7	WCL1 10	WCL1 14	WCL1 18	WCL1 21	WCL1 28	WCL2 2	WCL2 4	WCL2 7	WCL2 10	WCL2 14	WCL2 18	WCL2 21	WCL2 28	WCL2 35	WCL3 2	WCL3 4	WCL3 7	WCL3 10	WCL3 14	WCL3 18	WCL3 21	WCL3 24	WCL3 28
WCL1 1		0.69	0.48	0.40	0.37	0.38	0.31	0.37	0.35	0.57	0.39	0.35	0.29	0.30	0.28	0.30	0.31	0.30	0.60	0.40	0.35	0.34	0.33	0.31	0.32	0.32	0.31
WCL1 2	0.69		0.85	0.79	0.74	0.75	0.65	0.72	0.69	0.82	0.76	0.69	0.64	0.60	0.59	0.61	0.60	0.61	0.83	0.77	0.70	0.67	0.65	0.63	0.63	0.61	0.57
WCL1 4	0.48	0.85		0.97	0.94	0.93	0.88	0.91	0.89	0.66	0.89	0.86	0.83	0.79	0.78	0.80	0.78	0.79	0.66	0.91	0.88	0.85	0.82	0.80	0.80	0.77	0.70
WCL1 7	0.40	0.79	0.97		0.97	0.97	0.93	0.95	0.94	0.62	0.88	0.87	0.86	0.83	0.82	0.84	0.82	0.83	0.61	0.89	0.90	0.88	0.86	0.84	0.83	0.80	0.73
WCL1 10	0.37	0.74	0.94	0.97		0.97	0.96	0.96	0.95	0.55	0.84	0.87	0.86	0.85	0.85	0.85	0.85	0.85	0.56	0.85	0.89	0.88	0.87	0.85	0.85	0.82	0.76
WCL1 14	0.38	0.75	0.93	0.97	0.97		0.96	0.99	0.97	0.59	0.85	0.86	0.86	0.84	0.84	0.86	0.85	0.85	0.59	0.86	0.88	0.88	0.87	0.86	0.85	0.83	0.77
WCL1 18	0.31	0.65	0.88	0.93	0.96	0.96		0.98	0.98	0.48	0.78	0.83	0.85	0.85	0.87	0.87	0.86	0.86	0.50	0.80	0.86	0.87	0.87	0.87	0.87	0.84	0.78
WCL1 21	0.37	0.72	0.91	0.95	0.96	0.99	0.98		0.99	0.55	0.82	0.84	0.85	0.85	0.86	0.87	0.86	0.87	0.56	0.83	0.87	0.87	0.87	0.87	0.87	0.85	0.79
WCL1 28	0.35	0.69	0.89	0.94	0.95	0.97	0.98	0.99		0.52	0.80	0.83	0.85	0.84	0.85	0.87	0.86	0.87	0.54	0.82	0.86	0.87	0.87	0.87	0.87	0.86	0.80
WCL2 2	0.57	0.82	0.66	0.62	0.55	0.59	0.48	0.55	0.52		0.76	0.70	0.63	0.60	0.56	0.60	0.58	0.60	0.79	0.66	0.60	0.55	0.53	0.51	0.51	0.49	0.45
WCL2 4	0.39	0.76	0.89	0.88	0.84	0.85	0.78	0.82	0.80	0.76		0.96	0.93	0.88	0.87	0.89	0.86	0.87	0.65	0.90	0.88	0.84	0.81	0.79	0.78	0.74	0.67
WCL2 7	0.35	0.69	0.86	0.87	0.87	0.86	0.83	0.84	0.83	0.70	0.96		0.98	0.96	0.94	0.95	0.92	0.92	0.59	0.86	0.89	0.87	0.85	0.83	0.82	0.79	0.72
WCL2 10	0.29	0.64	0.83	0.86	0.86	0.86	0.85	0.85	0.85	0.63	0.93	0.98		0.98	0.97	0.97	0.95	0.94	0.54	0.83	0.88	0.88	0.87	0.86	0.85	0.83	0.77
WCL2 14	0.30	0.60	0.79	0.83	0.85	0.84	0.85	0.85	0.84	0.60	0.88	0.96	0.98		0.99	0.98	0.97	0.96	0.51	0.79	0.85	0.86	0.87	0.86	0.86	0.84	0.79
WCL2 18	0.28	0.59	0.78	0.82	0.85	0.84	0.87	0.86	0.85	0.56	0.87	0.94	0.97	0.99		0.99	0.97	0.96	0.49	0.78	0.85	0.86	0.87	0.87	0.87	0.85	0.79
WCL2 21	0.30	0.61	0.80	0.84	0.85	0.86	0.87	0.87	0.87	0.60	0.89	0.95	0.97	0.98	0.99		0.98	0.97	0.51	0.80	0.86	0.87	0.88	0.88	0.88	0.86	0.80
WCL2 28	0.31	0.60	0.78	0.82	0.85	0.85	0.86	0.86	0.86	0.58	0.86	0.92	0.95	0.97	0.97	0.98		0.98	0.50	0.77	0.84	0.85	0.87	0.86	0.86	0.85	0.80
WCL2 35	0.30	0.61	0.79	0.83	0.85	0.85	0.86	0.87	0.87	0.60	0.87	0.92	0.94	0.96	0.96	0.97	0.98		0.51	0.78	0.83	0.85	0.86	0.86	0.86	0.84	0.80
WCL3 2	0.60	0.83	0.66	0.61	0.56	0.59	0.50	0.56	0.54	0.79	0.65	0.59	0.54	0.51	0.49	0.51	0.50	0.51		0.73	0.66	0.63	0.61	0.59	0.60	0.59	0.58
WCL3 4	0.40	0.77	0.91	0.89	0.85	0.86	0.80	0.83	0.82	0.66	0.90	0.86	0.83	0.79	0.78	0.80	0.77	0.78	0.73		0.96	0.94	0.90	0.88	0.87	0.84	0.75
WCL3 7	0.35	0.70	0.88	0.90	0.89	0.88	0.86	0.87	0.86	0.60	0.88	0.89	0.88	0.85	0.85	0.86	0.84	0.83	0.66	0.96		0.98	0.96	0.95	0.93	0.90	0.82
WCL3 10	0.34	0.67	0.85	0.88	0.88	0.88	0.87	0.87	0.87	0.55	0.84	0.87	0.88	0.86	0.86	0.87	0.85	0.85	0.63	0.94	0.98		0.98	0.97	0.96	0.93	0.85
WCL3 14	0.33	0.65	0.82	0.86	0.87	0.87	0.87	0.87	0.87	0.53	0.81	0.85	0.87	0.87	0.87	0.88	0.87	0.86	0.61	0.90	0.96	0.98		0.99	0.98	0.96	0.89
WCL3 18	0.31	0.63	0.80	0.84	0.85	0.86	0.87	0.87	0.87	0.51	0.79	0.83	0.86	0.86	0.87	0.88	0.86	0.86	0.59	0.88	0.95	0.97	0.99		0.99	0.97	0.91
WCL3 21	0.32	0.63	0.80	0.83	0.85	0.85	0.87	0.87	0.87	0.51	0.78	0.82	0.85	0.86	0.87	0.88	0.86	0.86	0.60	0.87	0.93	0.96	0.98	0.99		0.98	0.93
WCL3 24	0.32	0.61	0.77	0.80	0.82	0.83	0.84	0.85	0.86	0.49	0.74	0.79	0.83	0.84	0.85	0.86	0.85	0.84	0.59	0.84	0.90	0.93	0.96	0.97	0.98		0.96
WCL3 28	0.31	0.57	0.70	0.73	0.76	0.77	0.78	0.79	0.80	0.45	0.67	0.72	0.77	0.79	0.79	0.80	0.80	0.80	0.58	0.75	0.82	0.85	0.89	0.91	0.93	0.96	

Pearson correlation: 0.28 – 0.99

D

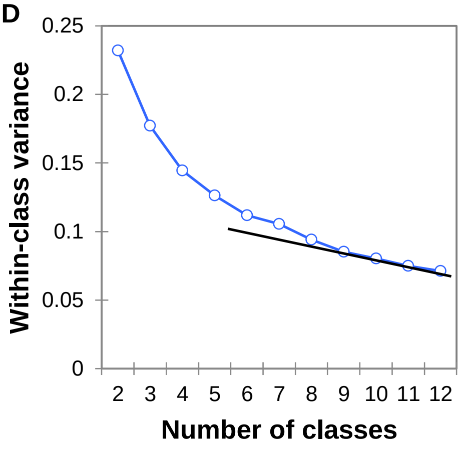

Figure S1. Related to Figure 1, Data From Three Biological Replicates are Concordant.

(A) Peptides and proteins quantified in all experiments in this manuscript. Of 712 human proteins quantified in experiment PM1, 475 were annotated by Gene Ontology ‘plasma membrane’, ‘cell surface’, ‘extracellular’ or ‘short GO’ (see Supplemental Experimental Procedures).

(B) Hierarchical cluster analysis of all proteins quantified in all three WCL experiments by 2 or more peptides.

(C) Matrix of Pearson correlations for all three WCL experiments. For every sample, fold change was calculated compared to the relevant uninfected control and then correlated to the fold change of every other sample.

(D) Plot of within-class variance against number of classes.

Figure S2

A **EBV-Encoded Protein Temporal Proteomic Profiles**

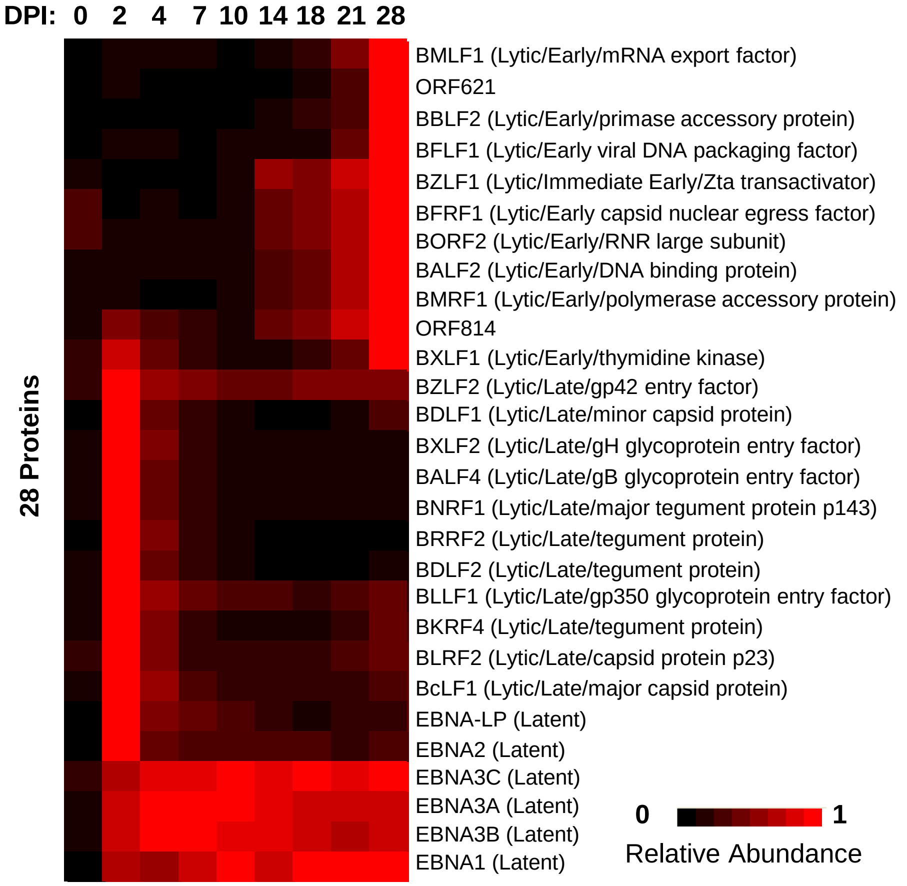

B

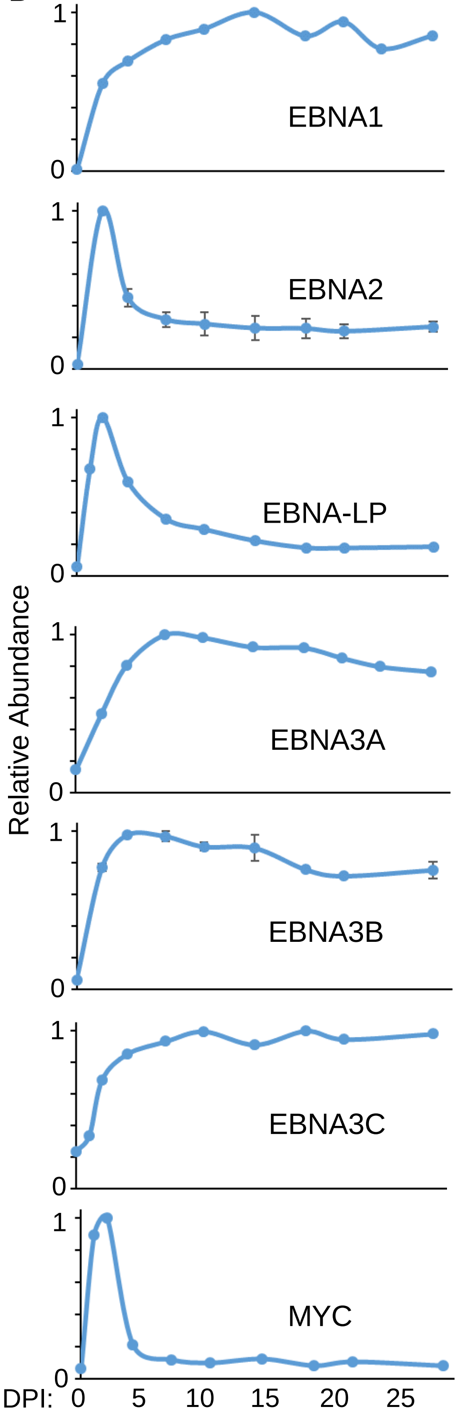

C **ORF814, Frame 5**

IITMEACPHIRYAFQNDKLLLQQASVGRLTLVNKTTILLRPMKTTTVDLGLYARPP
EGHGLMLWGSTSRPVTSHVGIIDPGYTGELRLILQNQRRYNSTLRPSELKIHLAA
FRYATPQMEEDKGPINHPQYPGDVGLDVSLPKDLALFPHQTVSVTLTVPPPSIP
HHRPTIFGRSGLAMQGILVKPCRWRRGGVDVSLTNFSDQTVFLNKYRRFCQLV
YLHKHHLTSFYSPHSDAGVLGPRSLFRWASCTFEEVPSLAMGDSGLSEALEGR
QGRGFGSSGQ

D **ORF621, Frame 4**

SSWRKDFNGRAFPLLLLAMRPKKDGLEDFLRLTPEIKKQLGSLVSDYCNVLNK
EFTAGSVEITLRSYKICKAFINEAKAHGREWGGLMATLNICNFWAILRNNRVRR
RAENAGNDACSIACPIVMRYVLDHLIVVTDRFFIQAPSNRVMIPATIGTAMYKLL
KHSRVRAYTYSKVLGVDRAAIMASGKQVVEHLNRMEKEGLLSSKFKAFCKWV
FTYPVLEEMFQTMVSSKTGHLTDDVKDVRALIKTLPRASYSSHAGQRSYVSGV
LPACLLSTKSKAVETPILVSGADRMDEELMGNDGGASHTEARYSESGQFHAFT
DELESLPSPTMPLKPGAQSADCGDSSSSSSDSGNSDTEQSEREEARAEAPRL
RAPKSRRTSRPNRGQTPCPSNAAEPEQPWIAAVHQESDERPIFPHPSKPTFLP
PVKRKKGLRDSREGMFLPKPEAGSAISDVFEGREVCQPKRIRPFHPPGSPWA
NRPLPASLAPTPTGPVHEPVGSLTPAPVPQPLDPAPAVTPEASHLLEDPDEET
SQAVKALREMADTVIPQKEEAAICGQMDLSHPPPRGHLDELTTTLESMTEDLN
LDSPLTPELNEILDTFLNDECLLHAMHISTGLSIFDTSLF

E

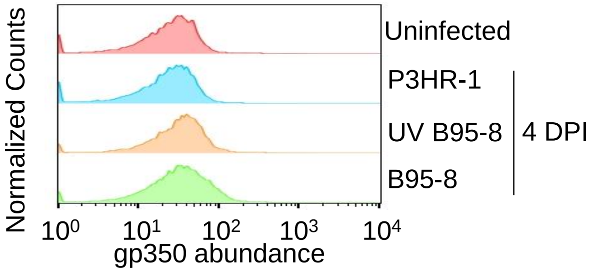

F

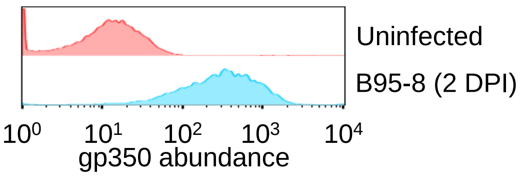

Figure S2. Related to Figure 1. Quantitative Temporal Analysis of EBV Proteins During Primary B-cell Transformation.

(A) Heatmap temporal plots of EBV proteins quantified in any of the three WCL replicates. Three distinct profiles were observed. Proteins have been annotated as latent or lytic cycle, expressed with immediate early, early or late kinetics, and associated function, if known.
(B) Temporal profiles of EBNA and MYC proteins.
(C) Amino acid sequence of newly identified EBV ORF814. Detected peptides are shown in green.
(D) Amino acid sequence of newly identified EBV ORF621. The detected peptide is shown in green.
(E) Flow cytometric analysis of cell surface EBV gp350 expression on primary B-cells infected with the indicated virus strains 4 DPI. Data shown is representative of n=3.
(F) Flow cytometric analysis of cell surface EBV gp350 expression on resting primary B-cells and cells infected with B95-8 virus 2 DPI. Data shown is representative of n=3.

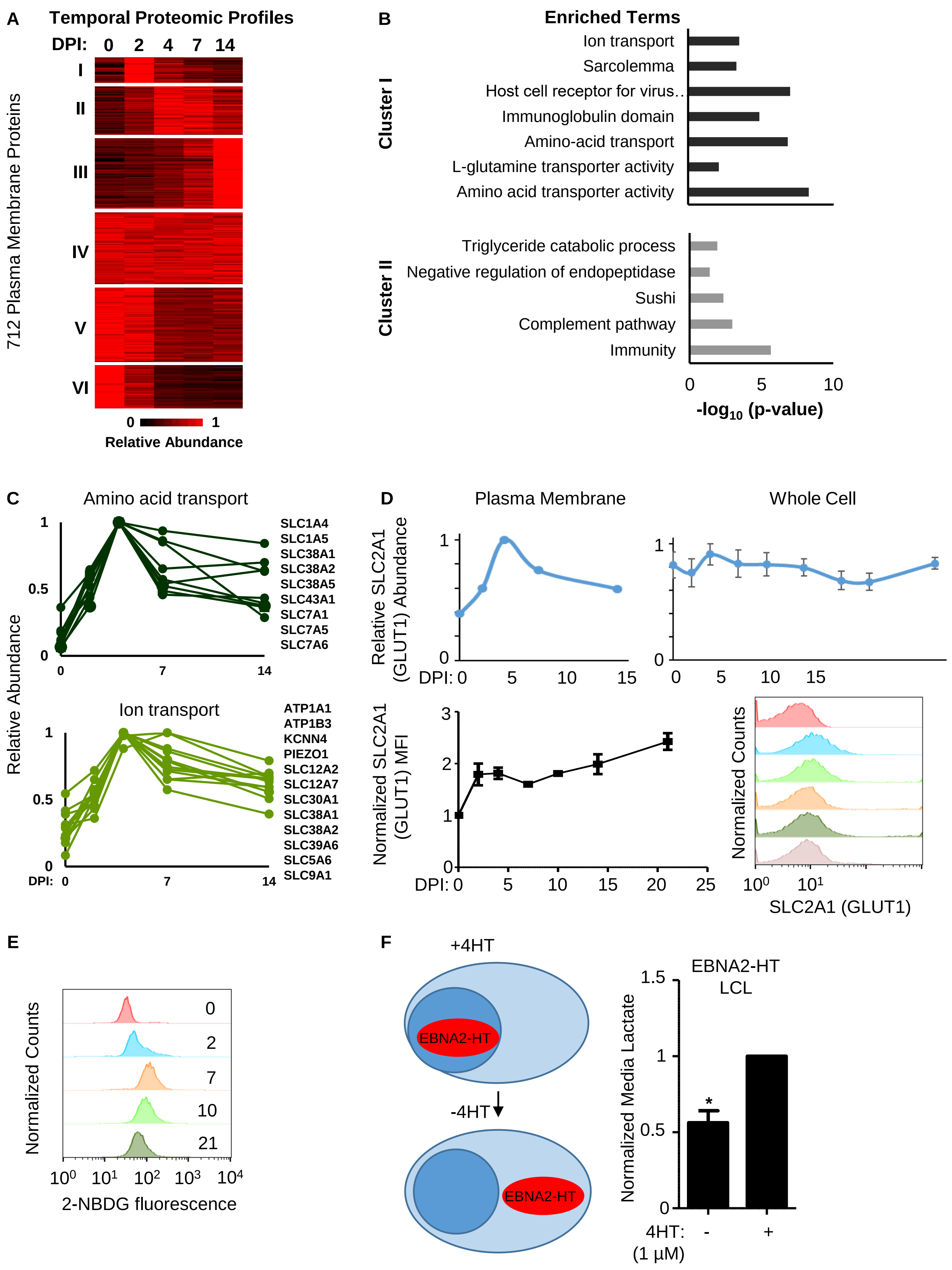
A
Temporal Proteomic Profiles
DPI: 0 2 4 7 14
712 Plasma Membrane Proteins
I
II
III
IV
V
VI
0 1
Relative Abundance
B
Enriched Terms
Cluster I
Ion transport
Sarcolemma
Host cell receptor for virus..
Immunoglobulin domain
Amino-acid transport
L-glutamine transporter activity
Amino acid transporter activity
Cluster II
Triglyceride catabolic process
Negative regulation of endopeptidase
Sushi
Complement pathway
Immunity
0 5 10
-log10 (p-value)
C
Amino acid transport
Relative Abundance
SLC1A4
SLC1A5
SLC38A1
SLC38A2
SLC38A5
SLC43A1
SLC7A1
SLC7A5
SLC7A6
Ion transport
ATP1A1
ATP1B3
KCNN4
PIEZO1
SLC12A2
SLC12A7
SLC30A1
SLC38A1
SLC38A2
SLC39A6
SLC5A6
SLC9A1
DPI: 0 7 14
D
Plasma Membrane
Whole Cell
Relative SLC2A1 (GLUT1) Abundance
DPI: 0 5 10 15
Normalized SLC2A1 (GLUT1) MFI
DPI: 0 5 10 15 20 25
Normalized Counts
SLC2A1 (GLUT1)
E
Normalized Counts
0
2
7
10
21
2-NBDG fluorescence
F
+4HT
EBNA2-HT
-4HT
EBNA2-HT
EBNA2-HT LCL
Normalized Media Lactate
*
4HT: (1 μM) - +

Figure S3, related to Figure 1. Modulation of PM Proteins Over Time During EBV-driven B-cell Transformation.

(A) Hierarchical cluster k-means analysis of the 712 B-cell plasma membrane (PM) proteins quantified in experiment PM1, by biotin labeling and streptavidin pulldown. K-means analysis was used to cluster B-cell PM proteins into 6 temporal categories.
(B) Functional enrichment analysis of EBV-induced PM proteins upregulated during EBV-induced transformation (clusters I-II).
(C) Temporal relative abundances of PM amino acid or ion transporters induced by EBV infection.
(D) Temporal traces of SLC2A1 (GLUT1) glucose transporter PM and WCL relative abundances. Normalized GLUT1 median fluorescent intensity (MFI) and flow cytometric histograms were also plotted against time for newly infected cells. Data shown are representative of n=3 biological replicates. Data for MFI show the mean ± SEM, n=3.
(E) Flow cytometry analysis of primary human B-cell glucose analogue 2-NBDG uptake at the indicated DPI. Histograms are representative of n=3 biological replicates.
(F) Normalized lactate secretion values from LCLs with conditional EBNA2-HT alleles, in the absence or presence of 4HT for 48 hours, as indicated. Mean + SEM are shown from n=3 biological replicates. *, $p<0.05$ (one-sample t-test).

Figure S4

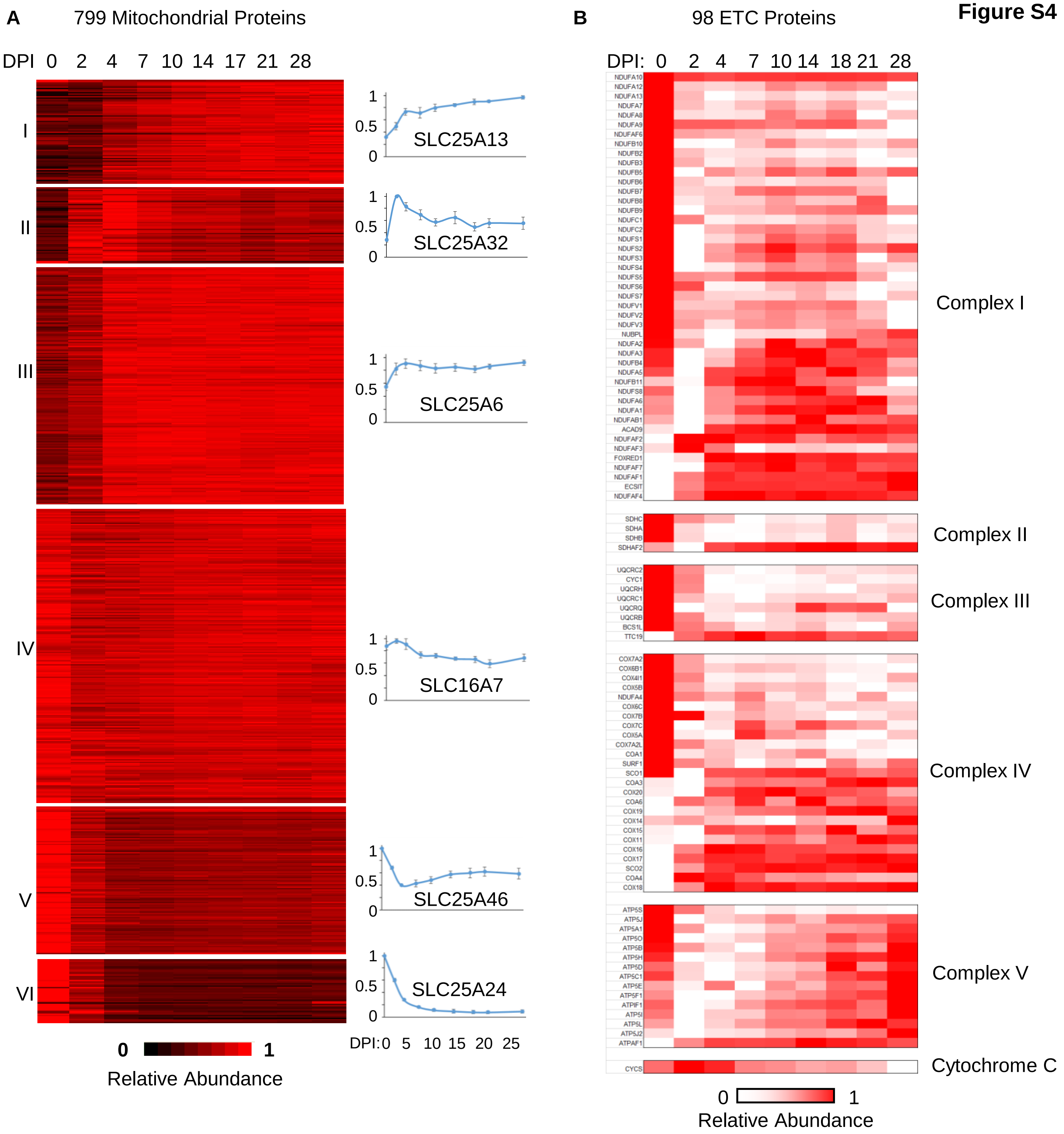

C **DAVID GO Analysis**

Term	Benjamini p-value
Mitochondrial carrier domain	4.19E-30
Transport	3.11E-16
Mitochondrial transport	2.26E-04
Adenine nucleotide translocator 1	0.01
Calcium-binding region	0.02
L-glutamate transport	0.04

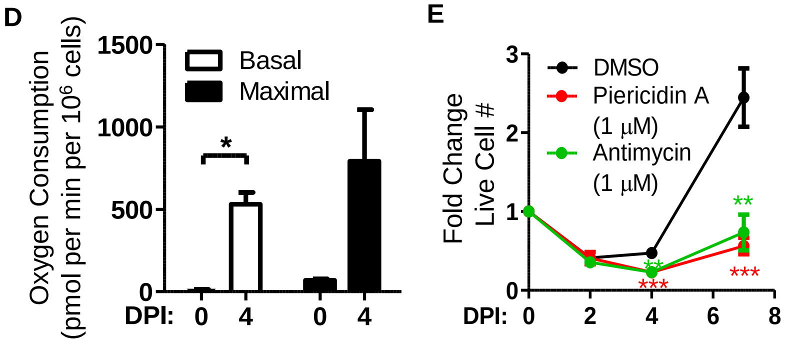

Figure S4, related to Figure 2. Mitochondrial Remodeling and Enhancement of ETC Activity Occur During EBV Growth Transformation.

(A) Hierarchical k-means cluster analysis of EBV-mediated remodeling of 799 MitoCarta-annotated proteins quantified by temporal proteomic analysis of viral primary B-cell growth transformation. Representative expression profiles of averaged relative abundances are shown to the right of each k-means cluster.
(B) Heat map of averaged relative abundances of mitochondrial electron transport chain components (98 quantified nuclear-encoded proteins) across the timecourse of EBV-mediated B-cell growth transformation.
(C) Functional enrichment analysis of mitochondrial pathways upregulated by EBV primary B-cell infection.
(D) Seahorse flux analysis of basal and maximal oxygen consumption rate in primary B-cells uninfected or at 4 DPI. Data show the mean + SEM, n=3. *, $p<0.05$ (paired one-tailed t-test).
(E) Growth curves of primary B-cells at the indicated DPI treated with DMSO vehicle, the complex I antagonist, piericidin A (1 µM), or the complex III antagonist, antimycin (1 µM). n=3 replicates. Data show the mean $\pm$ SEM, n=3. **, $p<0.01$; ***, $p<0.005$ (paired one-tailed t-test).

Figure S5

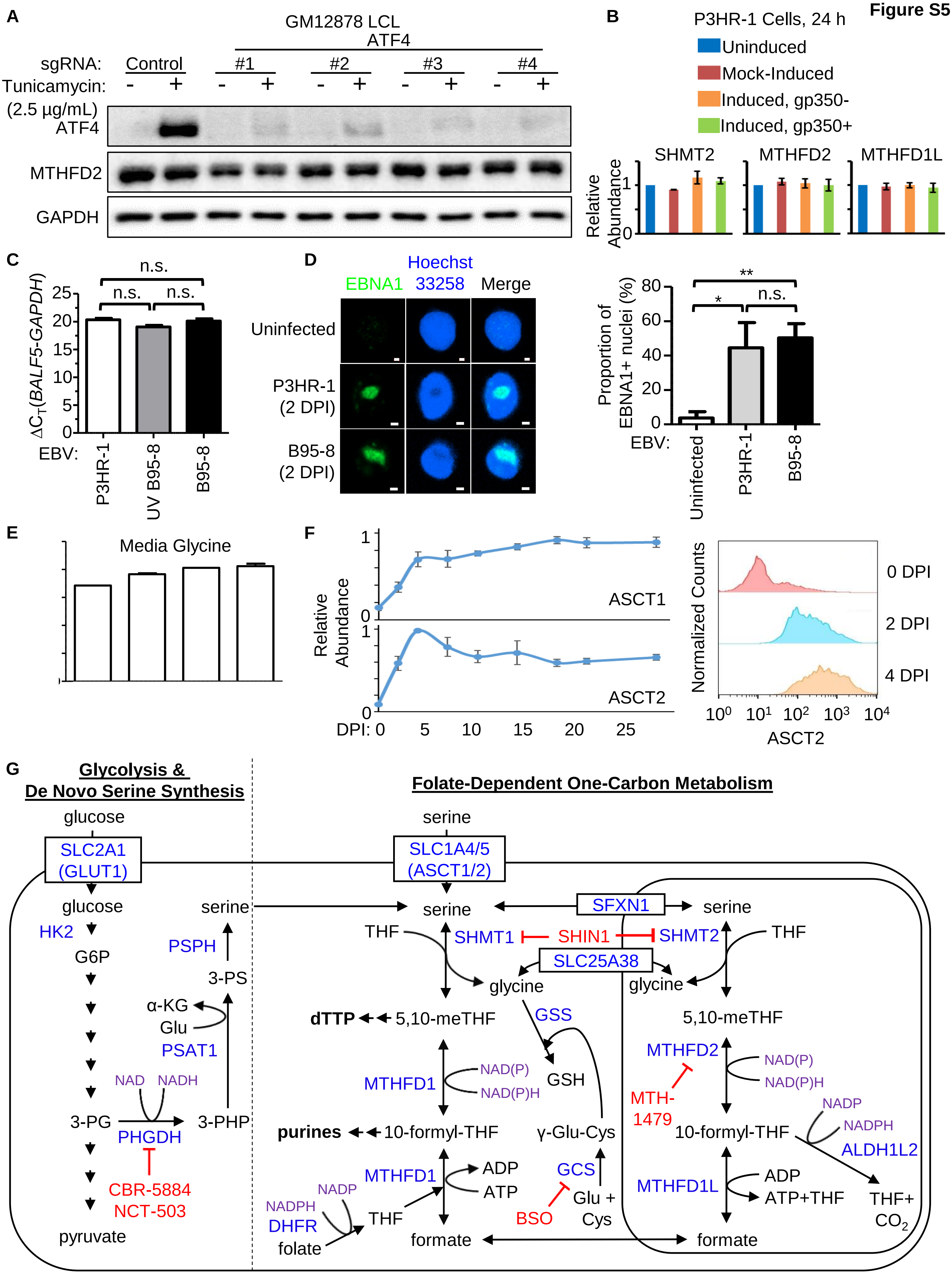
A
GM12878 LCL
ATF4
sgRNA: Control #1 #2 #3 #4
Tunicamycin: - + - + - + - + - +
(2.5 µg/mL)
ATF4
MTHFD2
GAPDH
B
P3HR-1 Cells, 24 h
Uninduced
Mock-Induced
Induced, gp350-
Induced, gp350+
SHMT2
MTHFD2
MTHFD1L
Relative Abundance
C
n.s.
n.s.
n.s.
ΔCT(BALF5-GAPDH)
EBV:
P3HR-1
UV B95-8
B95-8
D
EBNA1
Hoechst 33258
Merge
Uninfected
P3HR-1 (2 DPI)
B95-8 (2 DPI)
Proportion of EBNA1+ nuclei (%)
**
*
n.s.
EBV:
Uninfected
P3HR-1
B95-8
E
Media Glycine
F
Relative Abundance
ASCT1
ASCT2
DPI: 0 5 10 15 20 25
Normalized Counts
0 DPI
2 DPI
4 DPI
ASCT2
G
Glycolysis &
De Novo Serine Synthesis
Folate-Dependent One-Carbon Metabolism
glucose
SLC2A1 (GLUT1)
glucose
HK2
G6P
serine
PSPH
3-PS
α-KG
Glu
PSAT1
NAD
NADH
3-PG
PHGDH
3-PHP
CBR-5884
NCT-503
pyruvate
serine
SLC1A4/5 (ASCT1/2)
serine
SFXN1
serine
THF
SHMT1
SHIN1
SHMT2
THF
SLC25A38
glycine
glycine
dTTP
5,10-meTHF
GSS
5,10-meTHF
MTHFD1
NAD(P)
NAD(P)H
GSH
MTHFD2
NAD(P)
NAD(P)H
MTH-1479
purines
10-formyl-THF
γ-Glu-Cys
10-formyl-THF
NADP
NADPH
ALDH1L2
MTHFD1
ADP
ATP
GCS
MTHFD1L
ADP
ATP+THF
THF+ CO2
NADP
NADPH
DHFR
folate
THF
formate
BSO
Glu + Cys
formate

Figure S5, related to Figures 2-3. EBNA2 and MYC Regulate Serine Uptake, Synthesis and Metabolism.

(A) Immunoblot analysis of ATF4, MTHFD2 and load-control GAPDH expression in the Cas9+ EBV-transformed GM12878 lymphoblastoid cell line expressing non-targeting control or independent *ATF4*-targeting sgRNAs, and treated with DMSO vehicle or tunicamycin (2.5 µg/mL) as indicated. Representative of n=3.
(B) Relative abundances of the mitochondrial 1C enzymes SHMT2, MTHFD2 and MTHFD1L in parental P3HR-1 cells that do not have the ZHT/RHT conditional alleles but that were mock-induced by 4HT treatment for 24 hours (red), or in P3HR-1 cells that stably express conditional alleles of the EBV immediate early factors ZTA and RTA fused to modified estrogen receptor binding domain that were either uninduced (blue), or induced by 400 nM 4HT treatment for 24 hours and sorted into gp350- (orange) or lytic gp350+ (green) populations. Data are from (Ersing et al., 2017) and show the mean ±SEM of n=3 biological replicates.
(C) Quantitative PCR-based EBV genome copy number assays on primary B-cells newly infected with the indicated virus strains 24 hours post-infection. The difference in C_T values for *BALF5* and *GAPDH* was plotted for each condition. Data show the mean with SEM, n=3.
(D) Left: Representative EBNA1 immunofluorescence micrographs of primary B-cells newly infected with the indicated virus strains. White scale bars indicate a distance of 1 µm. Right: quantitation of EBNA1+ nuclei 2 DPI in ≥3 independent field of views for each condition. *, $p<0.05$; **, $p<0.01$ (unpaired two-tailed t-test).
(E) LC-MS measurement of media glycine levels in the absence or presence of primary human B-cells (seeded at 1 x 10^6 cells per mL) for 24 hours at the indicated DPI. Data shows the mean + SEM, n=3.
(F) Left: Temporal WCL profiles of ASCT1 and ASCT2 relative abundances at the indicated timepoints DPI. Data show the mean ± SEM, n=3. Right: Flow cytometry analysis of CD23+ primary B-cell ASCT2 PM levels at the indicated DPI. Representative of n=3.
(G) Schematic map of proteins, cofactors and metabolites of interest. Proteins are indicated in blue, cofactors are shown in purple and metabolites are labeled in black. Inhibitors used are also indicated in red.

Figure S6

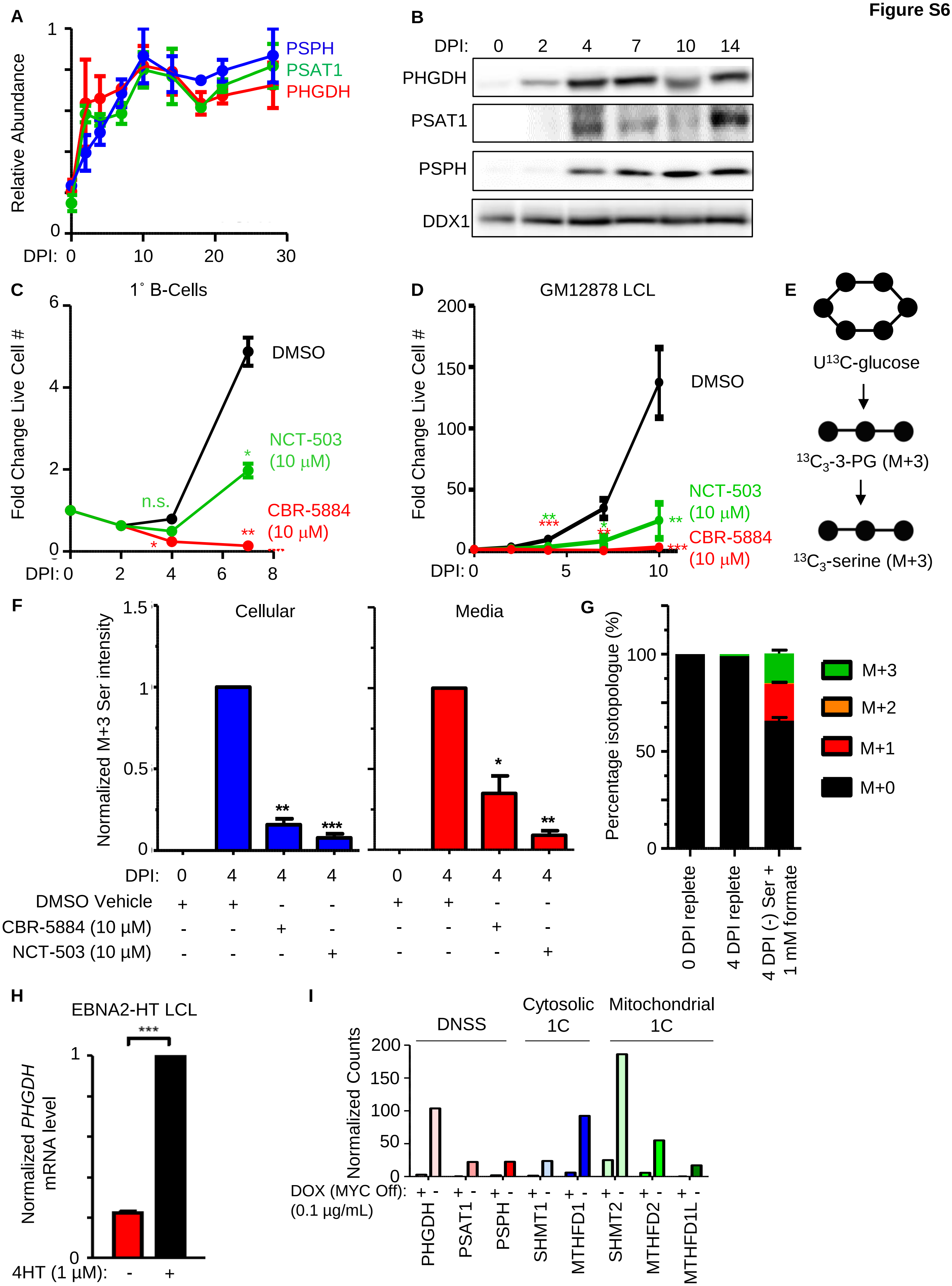
A
Relative Abundance
DPI: 0 10 20 30
PSPH
PSAT1
PHGDH
B
DPI: 0 2 4 7 10 14
PHGDH
PSAT1
PSPH
DDX1
C
1˚ B-Cells
Fold Change Live Cell #
DMSO
NCT-503 (10 µM)
CBR-5884 (10 µM)
n.s.
DPI: 0 2 4 6 8
D
GM12878 LCL
Fold Change Live Cell #
DMSO
NCT-503 (10 µM)
CBR-5884 (10 µM)
DPI: 0 5 10
E
U13C-glucose
13C3-3-PG (M+3)
13C3-serine (M+3)
F
Normalized M+3 Ser intensity
Cellular
Media
DPI: 0 4 4 4 0 4 4 4
DMSO Vehicle + + - - + + - -
CBR-5884 (10 µM) - - + - - - + -
NCT-503 (10 µM) - - - + - - - +
G
Percentage isotopologue (%)
M+3
M+2
M+1
M+0
0 DPI replete
4 DPI replete
4 DPI (-) Ser + 1 mM formate
H
EBNA2-HT LCL
Normalized PHGDH mRNA level
4HT (1 µM): - +
I
DNSS
Cytosolic 1C
Mitochondrial 1C
Normalized Counts
DOX (MYC Off): (0.1 µg/mL)
PHGDH
PSAT1
PSPH
SHMT1
MTHFD1
SHMT2
MTHFD2
MTHFD1L

Figure S6, related to Figure 3. De Novo Serine Synthesis (DNSS) is a Key EBV-infected B-cell Dependency Factor.

(A) Temporal profiles of the indicated DNSS enzymes at the indicated DPI. Data show the mean ± SEM, n=3.
(B) Immunoblot analysis of DNSS enzyme expression from whole cell lysates of primary B-cells at the indicated time points of EBV infection. Blots are representative of n=3 biological replicates.
(C) Growth curves of newly infected primary B-cells treated with either DMSO, PHGDH-selective antagonists CBR-5884 (10 µM) or NCT-503 (10 µM) . Data show the mean with SEM, n=3. *, $p<0.05$; **, $p<0.01$ (paired two-tailed t-test).
(D) Growth curves of GM12878 LCLs treated with either DMSO, PHGDH-selective antagonists CBR-5884 (10 µM) or NCT-503 (10 µM). Data show the mean with SEM, n=3. *, $p<0.05$; **, $p<0.01$; ***, $p<0.005$ (unpaired two-tailed t-test).
(E) Schematic illustrating metabolic tracing of $U^{13}C$-glucose in its conversion to ^{13}C-labeled serine.
(F) LC-MS detection of M+3 labeled serine in primary B-cell cellular and media extracts at the indicated time points and under the indicated conditions. DMSO treatment was used for normalization i.e. set as 1. Data show the mean with SEM, n=3. *, $p<0.05$; **, $p<0.01$; ***, $p<0.005$ (one-sample t-test).
(G) LC-MS detection of M+3 labeled serine in primary B-cell cellular extracts at the indicated time points post-infection and under replete or serine-deprived, formate-supplemented conditions.
(H) Quantitative PCR analysis of normalized *PHGDH* mRNA levels in 2-2-3 EBNA2-HT LCLs grown in the presence (permissive for growth) or absence (not permissive for growth) of 4-HT (1 µM) conditions for 48 hours. Data shows the mean with SEM, n=3. ***, $p<0.005$ (one-sample t-test).
(I) Normalized mRNA abundances in P493-6 LCLs grown under EBNA2 non-permissive conditions in the absence (exogenous *MYC* on) or presence of doxycycline (exogenous *MYC* off) (0.1 µg/mL) (data taken from (Lin et al., 2012).

Figure S7

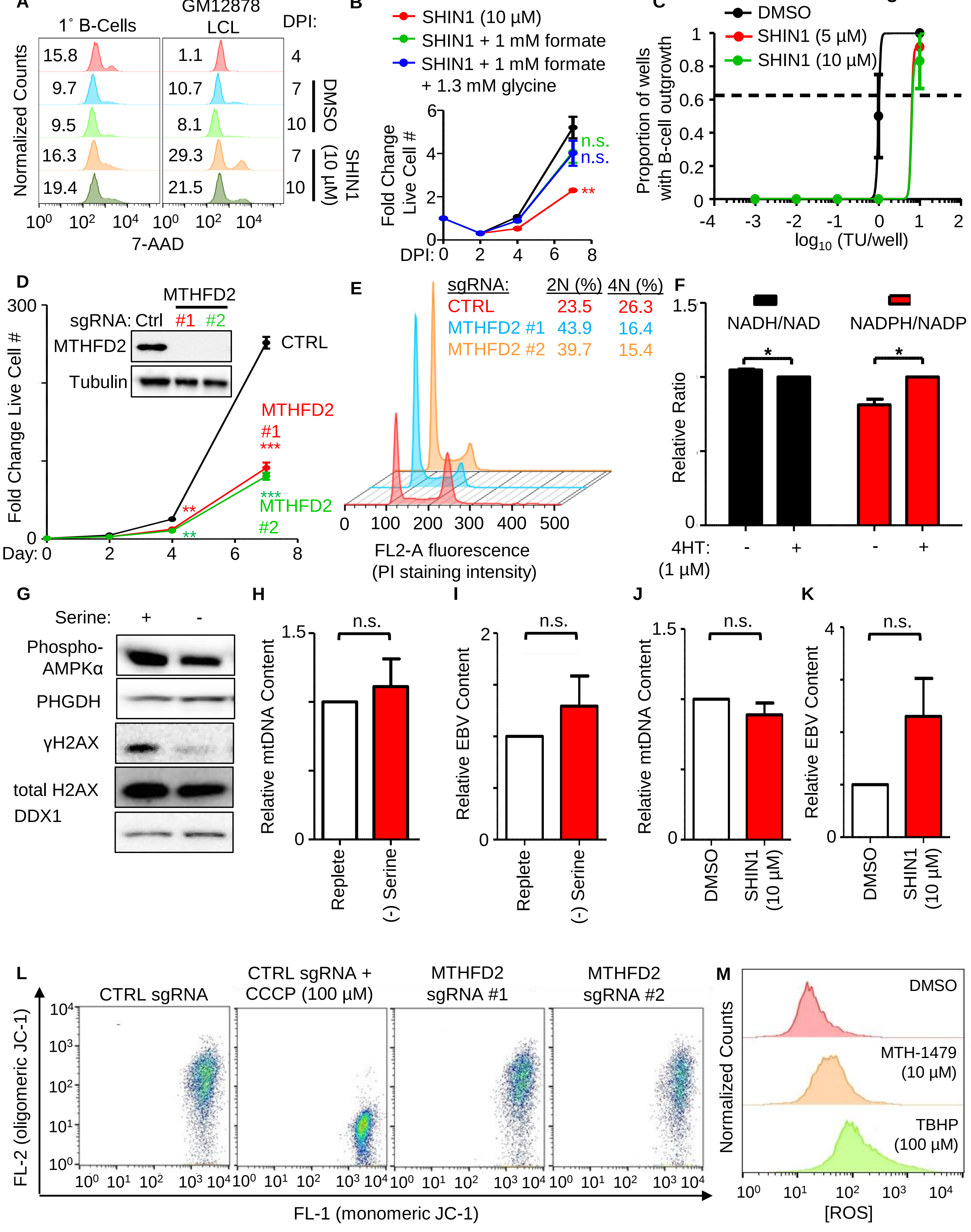
A
1˚ B-Cells
GM12878 LCL
DPI:
Normalized Counts
15.8
1.1
4
9.7
10.7
7
9.5
8.1
10
DMSO
16.3
29.3
7
19.4
21.5
10
SHIN1 (10 µM)
7-AAD
B
DMSO
SHIN1 (10 µM)
SHIN1 + 1 mM formate
SHIN1 + 1 mM formate + 1.3 mM glycine
Fold Change Live Cell #
n.s.
n.s.
**
DPI:
C
DMSO
SHIN1 (5 µM)
SHIN1 (10 µM)
Proportion of wells with B-cell outgrowth
log10 (TU/well)
D
MTHFD2
sgRNA: Ctrl #1 #2
MTHFD2
Tubulin
Fold Change Live Cell #
CTRL
MTHFD2 #1
***
**
***
**
MTHFD2 #2
Day:
E
sgRNA: 2N (%) 4N (%)
CTRL 23.5 26.3
MTHFD2 #1 43.9 16.4
MTHFD2 #2 39.7 15.4
FL2-A fluorescence (PI staining intensity)
F
NADH/NAD
NADPH/NADP
Relative Ratio
*
*
4HT: (1 µM)
G
Serine: + -
Phospho-AMPKα
PHGDH
γH2AX
total H2AX
DDX1
H
n.s.
Relative mtDNA Content
Replete
(-) Serine
I
n.s.
Relative EBV Content
Replete
(-) Serine
J
n.s.
Relative mtDNA Content
DMSO
SHIN1 (10 µM)
K
n.s.
Relative EBV Content
DMSO
SHIN1 (10 µM)
L
CTRL sgRNA
CTRL sgRNA + CCCP (100 µM)
MTHFD2 sgRNA #1
MTHFD2 sgRNA #2
FL-2 (oligomeric JC-1)
FL-1 (monomeric JC-1)
M
DMSO
MTH-1479 (10 µM)
TBHP (100 µM)
Normalized Counts
[ROS]

Figure S7, related to Figures 4-7. Inhibition of 1C Metabolism Adversely Affects NADPH Redox Balance but not Mitochondrial or Viral Genome Replication, the Adenylate Charge or Mitochondrial Membrane Potential.

(A) Apoptosis assays by 7-AAD staining of newly infected primary B-cells and LCLs treated with either DMSO or SHIN1 (10 µM). Data was collected at 4, 7 and 10 DPI. In-panel numbers indicate the average percentage of cells that were 7-AAD+. Data shown is representative of n=3.
(B) Growth curve analysis of newly infected cells treated with either DMSO, SHIN1 (10 µM) or SHIN1 with the indicated supplements. Data show the mean ± SEM, n=4. **, p<0.01 (paired two-tailed t-test).
(C) In vitro transformation assays of isolated primary human B cells treated continuously for 4 weeks with either DMSO or the indicated dose of SHIN1. Data show fitted non-linear regression curves with means ± SEM, n=3.
(D) Growth curve analysis of Cas9+ GM12878 LCLs following expression of non-targeting control versus independent *MTHFD2*-targeting sgRNAs. Data show the mean ± SEM, n=3. **, p<0.05; ***, p<0.005 (unpaired two-tailed t-test).
Inset: immunoblot analysis of MTHFD2 and tubulin load-control in Cas9+ GM12878 LCLs with the indicated sgRNAs. Shown are representative blots, n=3.
(E) PI staining of Cas9+ GM12878 LCLs expressing the indicated control or MTHFD2-targeting sgRNAs. Average percentages for 2N and 4N peaks across n=3 biological replicates are shown. Histograms are representative of n=3 biological replicates.
(F) Whole cell NADH/NAD ratios (black) and NADPH/NADP ratios (red) in 2-2-3 conditional EBNA2-HT LCLs grown in the absence or presence of 1 µM 4HT for 48 hours. Data show the mean + SEM of n=3 replicates. *, p<0.05 (one-sample t-test).
(G) Immunoblot analysis of WCL from primary B-cells at 4 DPI grown in replete or serine deficient media. Blots are representative of n=3 replicates.
(H) Relative mitochondrial DNA (mtDNA) content in primary B-cells 4 DPI grown in replete or serine-deficient media. Data show the mean + SEM, n=3. n.s., not significant (one-sample t-test).
(I) Relative EBV DNA content in primary B-cells 4 DPI grown in replete or serine-deficient media. Data show the mean + SEM, n=3. n.s., not significant (one-sample t-test).
(J) Relative mtDNA content in primary B-cells at 4 DPI grown in complete media with DMSO or SHIN1 (10 µM). Mean + SEM from n=3. n.s., not significant (one-sample t-test).
(K) Relative EBV DNA content in primary B-cells at 4 DPI grown in complete media with DMSO or SHIN1 (10 µM). Mean + SEM from n=3. n.s., not significant (one-sample t-test).
(L) JC-1 staining of Cas9+ GM12878 LCLs transduced with either control sgRNA or one of two *MTHFD2*-targeting sgRNAs. CCCP treatment was performed as a positive control. Contour plots shown are representative of n=2 replicates.
(M) Flow cytometry of DMSO- and MTH-1479 (10 µM)-treated cells at 7 DPI stained with DCFDA for intracellular ROS quantitation. TBHP (100 µM) was used as a positive control. Histogram shown is representative of n=2 replicates.
